# Gut microbiome dysbiosis in chronic lung disease: a systematic review and meta-analysis

**DOI:** 10.3389/fcimb.2025.1554846

**Published:** 2025-10-10

**Authors:** Juan Pan, Yanjie Zhang, Songlin Qiu, Shuotao Peng, Haixin Kang, Tao-Hsin Tung, Bo Shen

**Affiliations:** ^1^ Department of Clinical Laboratory, Taizhou Hospital of Zhejiang Province Affiliated to Wenzhou Medical University, Linhai, China; ^2^ Evidence-based Medicine Center, Taizhou Hospital of Zhejiang Province, Wenzhou Medical University, Linhai, China

**Keywords:** chronic lung disease, gut, microbiome, diversity, dysbiosis

## Abstract

**Objectives:**

Recent data suggest that the gut–lung axis plays a role in the development of lung disease. However, the potential association between the gut microbiota and chronic lung disease (CLD) remains unclear. This study aimed to investigate changes in the gut microbiome in patients with CLD.

**Methods:**

We searched PubMed, Web of Science, Cochrane, Embase, China National Knowledge Infrastructure, Wanfang, and VIPC databases from inception to August 1, 2024. The inclusion criteria involved studies that reported on gut flora in patients with CLD. Two independent reviewers used standardized methods to search, screen, and code included studies. Publication bias was analyzed using Egger’s test. Changes in the gut microbiome were assessed through α-diversity, β-diversity, and changes/differences in relative abundance, and results were evaluated using a random-effects model.

**Results:**

A total of 27 studies were included: 21 on chronic obstructive pulmonary disease (COPD; 1,273 patients and 6,718 healthy controls [HCs]), six on asthma (559 patients and 5,310 HCs), one on COPD and asthma combined, and one on pulmonary cystic fibrosis. Compared with HCs, α-diversity was decreased in patients with COPD (Shannon index: standard mean difference [SMD] = −0.38; 95% confidence interval [CI], −0.76 to 0.00, *I^2^* = 72%; n=7). In COPD, Bacteroides was increased (SMD = 0.76; 95% CI, 0.00 to 1.52; *I^2^* = 91%; n=4), while Bifidobacterium (SMD = −0.88; 95% CI, −1.39 to −0.37; *I^2^* = 88%; n=5) and Lactobacillus (SMD = -0.73; 95% CI, -1.00 to -0.46; *I^2^* = 66%; n=5) were decreased. No difference was found in Shannon and Simpson diversity indexes between patients with asthma and HCs.

**Conclusion:**

The gut microbiota of patients with COPD is imbalanced, and the abundance of probiotics is lower than in healthy individuals. Further exploration of the potential role of probiotics in COPD may provide promising targets for treatment.

**Systematic review registration:**

https://www.crd.york.ac.uk/PROSPERO/, identifier CRD42022378296.

## Introduction

Chronic lung disease (CLD) affects many individuals and is a major cause of morbidity and mortality. The most common CLDs are asthma and chronic obstructive pulmonary disease (COPD), and inflammatory responses can influence the onset and progression of these conditions. Evidence suggests that the gut microbiota impacts immune inflammation (Van de Wiele et al., 2016). The human body hosts a wide range of microorganisms that act synergistically with the immune system and exert protective effects. Smoking, antibiotic use, and steroid use—factors strongly associated with COPD and asthma—can disrupt the balance of the lung microbiota (Song et al., 2024). Alterations in the lung microbiota play an important role in disease progression in patients with COPD and asthma. Increases in *Haemophilus influenzae, Pseudomonas aeruginosa, Aspergillus* spp., and *Streptococcus* can contribute to disease exacerbation (Tangedal et al., 2019; Barcik et al., 2020; Russo et al., 2022; Zhu and Chang, 2023). Oropharyngeal bacteria can exacerbate lung microbial dysbiosis and enhance lung damage caused by *P. aeruginosa* by affecting its genomic activity or by translocating to the respiratory system (Duan et al., 2003; Wen et al., 2022). Chai et al (Chai et al., 2021), Avalos-Fernandez et al (Avalos-Fernandez et al., 2022), and Najafi et al. (2021) conducted meta-analyses on the relationship between lung diseases and the lung microbiota and found that the pulmonary microbiota of patients with COPD, asthma, and cystic fibrosis was abnormal. The critical role of the microbiota in patients with CLDs is evident.

The gut is the largest immune organ in the body. With the development of detection technologies such as second-generation sequencing, there has been an explosion of research on the diversity and relative abundance of the gut microflora. Gut microbiota contribute to nutrient metabolism and regulate immunity (Xiao et al., 2021). However, various factors can disrupt their diversity and abundance, leading to imbalances in gut ecology (Cho and Blaser, 2012). Gut dysbiosis is characterized by the loss of symbiotic relationships within the microbial ecosystem, resulting from changes in composition, overgrowth of potentially pathogenic microorganisms, and reduced biodiversity of bacterial species (De Nuccio et al., 2022). Moreover, gut microbes and their metabolites can affect the function of other organs, such as the liver, brain, and intestine (Hebbandi Nanjundappa et al., 2022).

The gastrointestinal tract and lungs share a common embryonic origin, and dysregulation of respiratory and intestinal immune systems are interrelated, suggesting a “gut–lung axis” linking intestinal microecology to lung disease (Mjösberg and Rao, 2018). COPD has become the third leading cause of death worldwide, and its prevalence is likely to increase (Krumina et al., 2022). COPD coexists with chronic gastrointestinal diseases, and patients with COPD are at higher risk of developing inflammatory bowel disease than healthy individuals (Budden et al., 2017). The intestinal flora of patients with COPD stages III–IV showed a significant increase in Firmicutes and a decrease in Bacteroidaceae and Fusobacteriaceae compared to those in stages I–II (Li et al., 2021; Chiu et al., 2022). Moreover, long-term pulmonary ventilatory dysfunction due to COPD impacts gas exchange and oxygen supply, which may alter the intestinal microenvironment and thereby affect intestinal flora. Gut microbiota and their metabolites, in turn, induce the production of immune cells and cytokines that enter systemic circulation through the blood and lymphatic systems to regulate immune and inflammatory responses in the lung. For example, symbiotic gut microbiota enhance host defense mechanisms against bacterial pneumonia by increasing IL-17A expression levels and upregulating granulocyte–macrophage colony-stimulating factor signaling in the lung (Brown et al., 2017). In addition, the gut microbiota can influence the function of nucleotide-binding oligomerization domain (NOD)-like receptor (NLR) family pyrin domain-containing protein (NLRP3) (Donovan et al., 2020; Qu et al., 2022), a core component of the inflammasome that promotes secretion of inflammatory factors. This secretion, in turn, may contribute to airway inflammation in chronic lung conditions such as COPD and asthma (Wang et al., 2018; Donovan et al., 2020; Qu et al., 2022). Metabolites of intestinal microorganisms, such as short-chain fatty acids, trimethylamine N-oxide (TMAO), and lysine, can also increase the secretion of pro-inflammatory factors in patients with COPD or CLDs such as asthma (Fujimura and Lynch, 2015; De Nuccio et al., 2022; Kayongo et al., 2022; Krumina et al., 2022). Inflammation in the intestinal tract can damage physiological barriers, resulting in bacterial translocation and dissemination of bacterial lipopolysaccharides from the gut throughout the body, which activate complement and promote inflammation (Rittirsch et al., 2008; Al Bander et al., 2020; Lim et al., 2023). The relationship between pediatric respiratory diseases (Alcazar et al., 2022), cystic fibrosis (Caley et al., 2023) and the intestinal microbiota has also been analyzed, with studies finding altered intestinal flora in patients with CLDs (Chen, 2018; Yong et al., 2020; Wu et al., 2021). However, the results are inconsistent, and detailed characteristics remain unclear.

Systematic reviews and meta-analyses are effective methods to determine the consistency of multiple studies. To our knowledge, no such studies have been conducted on alterations in the gut microbiota of patients with CLDs. Therefore, our study aimed to synthesize cumulative evidence on the association between the gut microbiota and CLDs and to provide potential therapeutic strategies for the treatment and management of CLD.

## Methods

The protocol of this review was pre-registered with PROSPERO (CRD42022378296). We followed the Preferred Reporting Items for Systematic Reviews and Meta-Analyses (PRISMA) reporting guidelines (Hutton et al., 2015). A defined framework, PO (P = population and O = outcomes), was used to determine the eligibility criteria of the articles. The study population comprised patients with CLDs, including COPD, asthma, emphysema, bronchitis, pulmonary hypertension, cystic fibrosis, idiopathic pulmonary fibrosis, and other chronic respiratory disorders. The outcomes for the review were α-diversity, β-diversity, and the abundance of gut flora in patients with CLDs.

### Search strategy

Extensive literature searches were conducted in PubMed, Web of Science, Cochrane, Embase, CNKI, Wanfang, and VIPC, using both MeSH terms and free-text keywords. All literature from database inception to 1 August 2024 was screened. No language restrictions were applied, and the search process is presented in **Supplementary Table 1**.

### Study selection and quality assessment

Literature screening and quality assessment were performed by Pan Juan and Zhang Yanjie. Any disagreements were resolved through consultation with a third investigator (Shen Bo). To observe changes in the intestinal flora of patients with CLDs, inclusion criteria were set as follows: studies analyzing and reporting α-diversity, β-diversity, or abundance of gut flora in patients with CLDs; and studies involving adult patients (aged ≥18 years). Exclusion criteria were studies without available full text, those without healthy controls (HCs), and reviews, meta-analyses, case reports, or letters to the editor. Risk of Bias in Non-randomized Studies of Exposures (ROBINS-E) was used to assess the quality of included literature (Stang, 2010).

### Data extraction

Data were extracted independently by two researchers, Juan Pan and Yanjie Zhang, and confirmed by a third researcher (Bo Shen) using a uniform extraction form. Extracted data included publication details, α-diversity, β-diversity, and differentially abundant microbial taxa. α-diversity provides a summary of microbial communities in a single sample and assesses richness (number of taxa) and evenness (distribution of community abundance). Community richness was estimated using Chao1 (total number of species in the sample) and ACE (abundance-based coverage estimation). Shannon’s index and Simpson’s index were used to reflect both richness and evenness of species in the sample. β-diversity measures inter-individual diversity and assesses similarity between communities and other samples analyzed.

### Data processing

The mean (M) and standard deviation (SD) of α-diversity indexes and relative abundance of microbial taxa were extracted. For studies reporting non-normally distributed data, when the median and interquartile range were provided, values were converted to M and SD using a web-based tool (https://www.math.hkbu.edu.hk/~tongt/papers/median2mean.html). When necessary, numerical data were extracted from bar graphs using WebPlotdigitizer (V.4.4) (Drevon et al., 2017). Standardized mean differences (SMDs) and 95% confidence intervals (CIs) were calculated for the above indicators in patients with CLD and HCs. When a study reported results by subgroups (except case–controls and respiratory diseases), they were combined into a single group. Two-sided P values <0.05 were considered statistically significant. Meta-analyses and sensitivity analyses were conducted using the R packages meta and metafor (version 4.2-1). Heterogeneity was indicated by *I^2^*, and when *I^2^* > 50% a random-effects model was used, otherwise a fixed-effects model was applied. Results are presented as forest plots. Publication bias was assessed using Egger’s test, with P >0.05 indicating no publication bias. A meta-analysis could not be performed for β-diversity because complete data were not available in the included studies. Results are summarized in **Supplementary Table 4**.

### Patient and public involvement

Patients and the public were not involved in this study.

## Results

### Study selection

**Figure 1:**
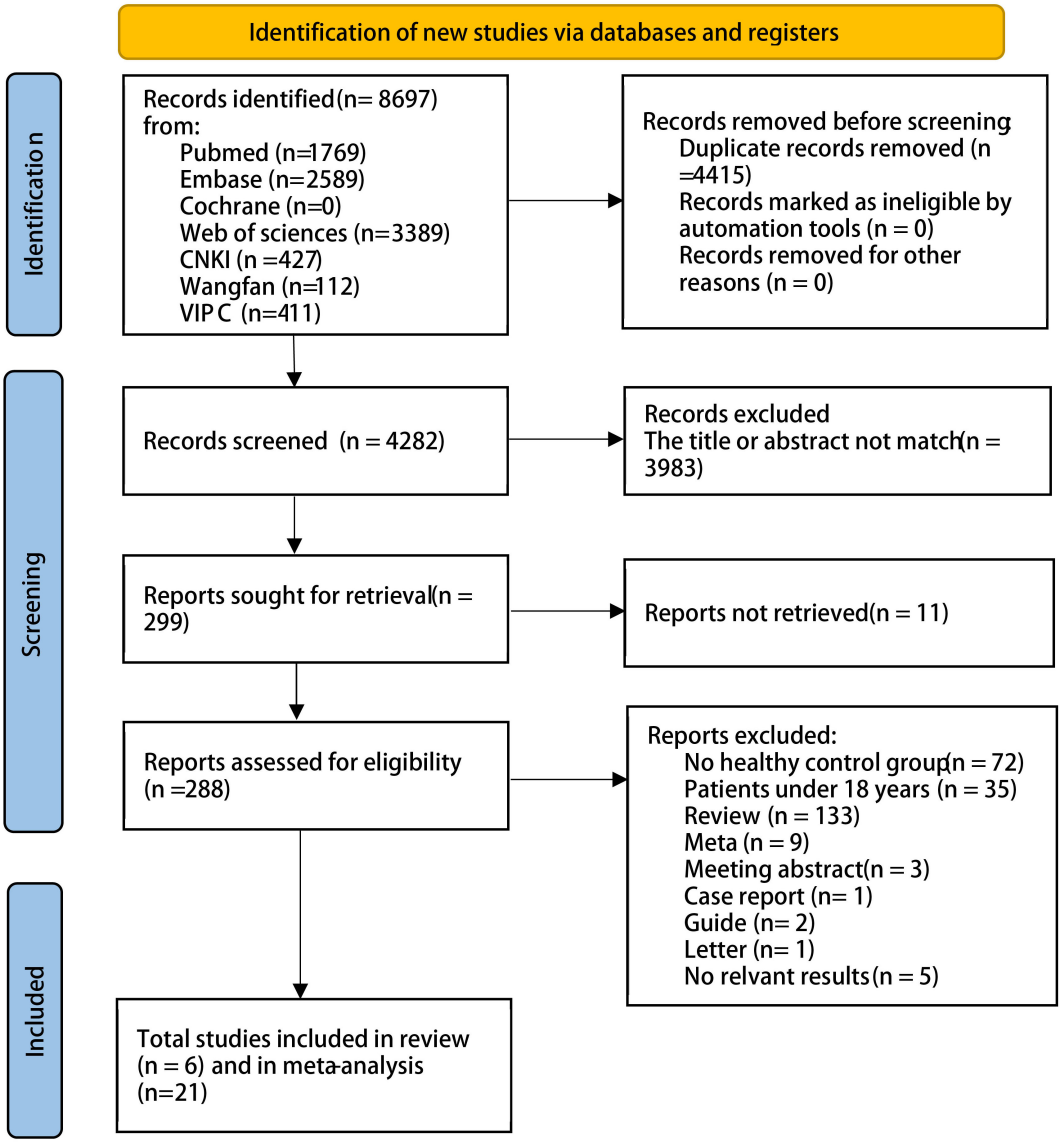
PRISMA study flow chart.

We searched English- and Chinese-language databases based on keywords and identified 8,697 articles as of 1 August 2024. A total of 27 articles met the inclusion and exclusion criteria: 20 on COPD, five on asthma, one on both COPD and asthma, and one on pulmonary cystic fibrosis. The detailed process is shown in **Figure 1**; **Supplementary Table 2**. In total, there were 1,273 patients with COPD and 6,718 HCs, and 559 patients with asthma and 5,310 HCs.

### Study characteristics

The characteristics of the 27 included studies are presented in **Supplementary Table 2**. The studies were published between 2013 and 2024. Most were conducted in China, and definitions of CLD were described in detail. Some studies of patients with CLD were excluded to avoid including those who had received antibiotics before specimen collection. All samples were collected from feces and mainly stored at −80 °C. Three studies performed bacterial cultures, 16 employed 16S rRNA analysis, five utilized metagenomic sequencing for bacterial detection, and three did not provide detailed descriptions. The risk of bias assessment is presented in **Supplementary Table 3**. Almost all studies were at risk of bias, primarily owing to confounding bias; however, few showed a high risk of selection bias.

### α-diversity of intestinal flora in patients with COPD

**Figure 2:**
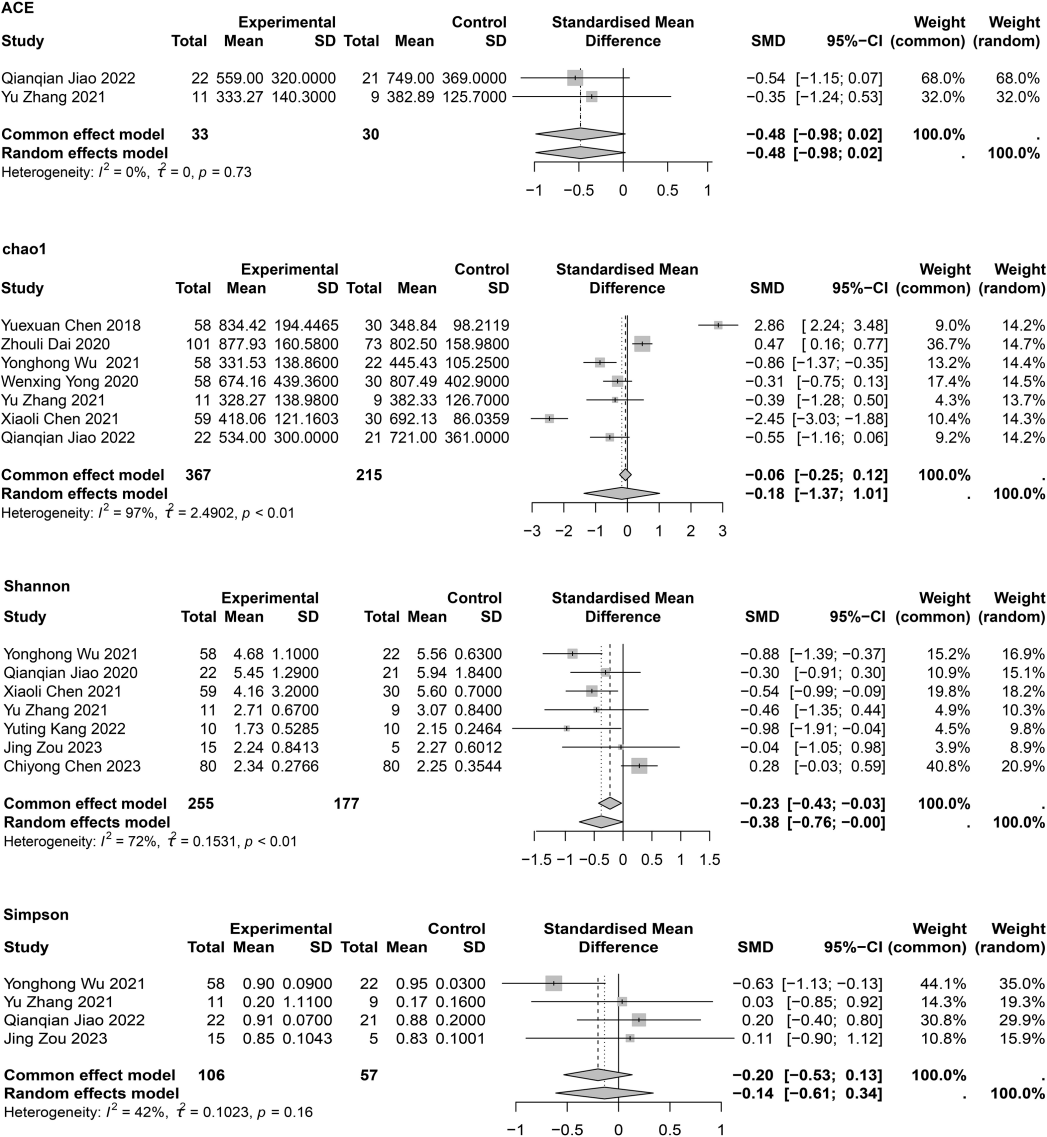
Meta-analysis of gut microbiome diversity in COPD patients.

The α-diversity of intestinal flora included both community richness and evenness. Richness was mainly assessed using Chao1 and ACE indexes, while evenness was assessed using the Shannon and Simpson indexes. Meta-analyses of α-diversity are presented in **Figure 2**. Publication bias assessments are shown in **Supplementary Table 6**, with no significant publication bias detected.

Regarding richness, two studies (Chen, 2021; Wu et al., 2021) reported lower Chao1 indexes in patients with COPD compared to HCs, two studies (Chen, 2018; Dai, 2020) showed contradictory results, and three studies (Yong et al., 2020; Zhang, 2021; Jiao Qianqian et al., 2022) showed no significant differences between groups. One cohort study (Liu et al., 2022) also found no differences in α-diversity between patients with COPD and HCs, possibly attributable to specific microbial taxa rather than the overall microbial community. The meta-analysis showed no significant differences between groups (SMD = −0.118; 95% CI, −1.37 to 1.01; *I^2^* = 99%, τ^2^ = 2.4902). Owing to high heterogeneity, the quality of evidence for this outcome was interpreted as very low. Sensitivity analysis showed that after removing Yuexuan Chen’s study (Chen, 2018), heterogeneity was slightly reduced, but Chao1 values remained unchanged between groups (**Supplementary Figure 1**). A subgroup analysis was also conducted to investigate the impact of traditional Chinese medicine on intestinal flora in COPD, and results showed no significant differences in Chao1 between patients with COPD and HCs regardless of treatment (**Supplementary Figure 2**). Two studies (Zhang, 2021; Jiao Qianqian et al., 2022) reported ACE indexes (SMD = −0.48; 95% CI, −0.98 to 0.02; *I* ^2^ = 0%; τ^2^ = 0). No statistical differences in species richness were observed between groups.

Regarding evenness, seven studies reported the Shannon index; three (Chen, 2021; Wu et al., 2021; Kang et al., 2022) (Chen, 2021; Wu et al., 2021; Kang et al., 2022) showed lower gut microorganism diversity in patients with COPD compared to HCs, while four (Zhang, 2021; Jiao Qianqian et al., 2022; Chen C., 2023; Zou et al., 2023) showed no difference. The meta-analysis indicated a significant difference (SMD = −0.38; 95% CI, −0.76 to −0.00; *I* ^2^ = 72%; τ^2^ = 0.1531), with reduced species diversity in patients with COPD. Sensitivity analysis showed that after removing Chiyong Chen’s study (Chen C., 2023), heterogeneity decreased to *I* ^2^ = 0% and the meta-analysis still showed a significant difference (SMD = −0.58; 95% CI, −0.83 to −0.32; *I* ^2^ = 0%; τ^2^ = 0) (**Supplementary Figure 1**). However, for the four (Wu et al., 2021; Zhang, 2021; Jiao Qianqian et al., 2022; Zou et al., 2023) provided meta-analysis of the Simpson indexes found no statistically significant difference (SMD = −0.20; 95% CI, −0.53 to 0.13; I² = 42%; τ² = 0.1023). In summary, the findings suggest that species diversity in patients with COPD differs from that of HCs.

### α-diversity of intestinal flora in patients with asthma

**Figure 3:**
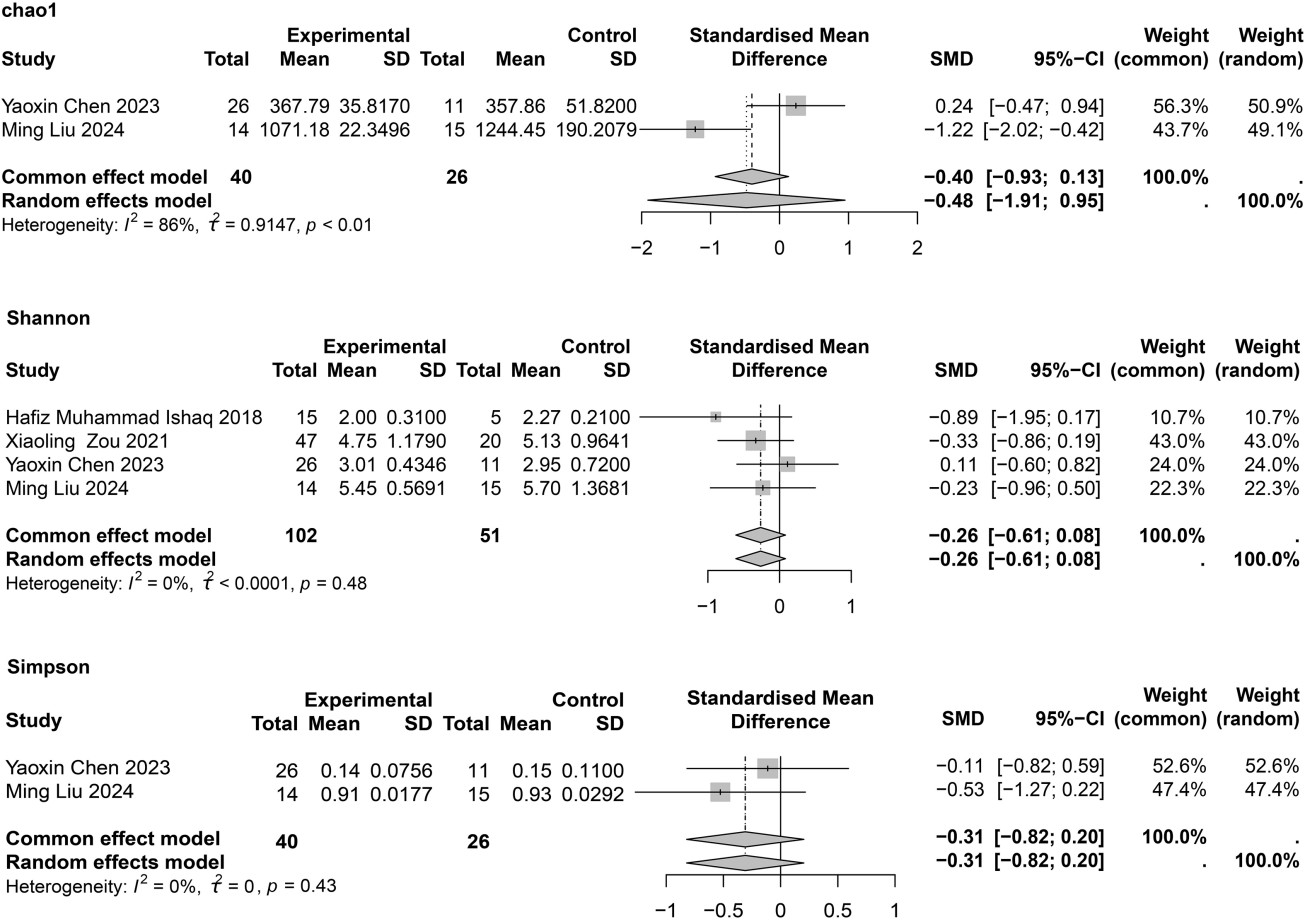
Meta-analysis of gut microbiome diversity in asthma patients.

For asthma, four studies (Ishaq et al., 2018; Zou et al., 2021; Chen Y., 2023; Ming et al., 2024) reported the Shannon index, and each showed no significant differences between patients with asthma and HCs. In the meta-analysis, no significant differences were found between the two groups (SMD = −0.26; 95% CI, −0.61 to 0.08; *I* ^2^ = 0%; τ^2^ < 0.0001) (**Figure 3**). Only two studies (Chen Y., 2023; Ming et al., 2024) provided the Simpson index, and we found no statistical difference between the two groups (SMD = −0.31; 95% CI, −0.82 to 0.20; *I* ^2^ = 0%; τ^2^ = 0). Egger’s test for publication bias is presented in **Supplementary Table 6**, and no significant publication bias was observed.

### β-diversity of intestinal flora in patients with CLDs

In the 27 included articles, 13 articles (Chen, 2018; Bowerman et al., 2020; Dai, 2020; Yong et al., 2020; Chen, 2021; Li et al., 2021; Wu et al., 2021; Zhang, 2021; Kang et al., 2022; Yimeng et al., 2022; Chen C., 2023; Zou et al., 2023; Yan et al., 2024)provided comparisons of β-diversity between patients with COPD and HCs (**Supplementary Table 4**). Most studies used principal component analysis; 12 showed significant differences between the COPD and HC groups, and one (Yimeng et al., 2022) showed no difference, indicating that the intestinal flora of patients with COPD is more likely to differ from that of healthy individuals. Three studies (Zou et al., 2021; Chen Y., 2023; Ming et al., 2024) provided comparisons of β-diversity between patients with asthma and HCs, all of which showed differences between the two groups, indicating that the intestinal flora of patients with asthma may differ from that of healthy individuals.

### Phylum levels

**Figure 4:**
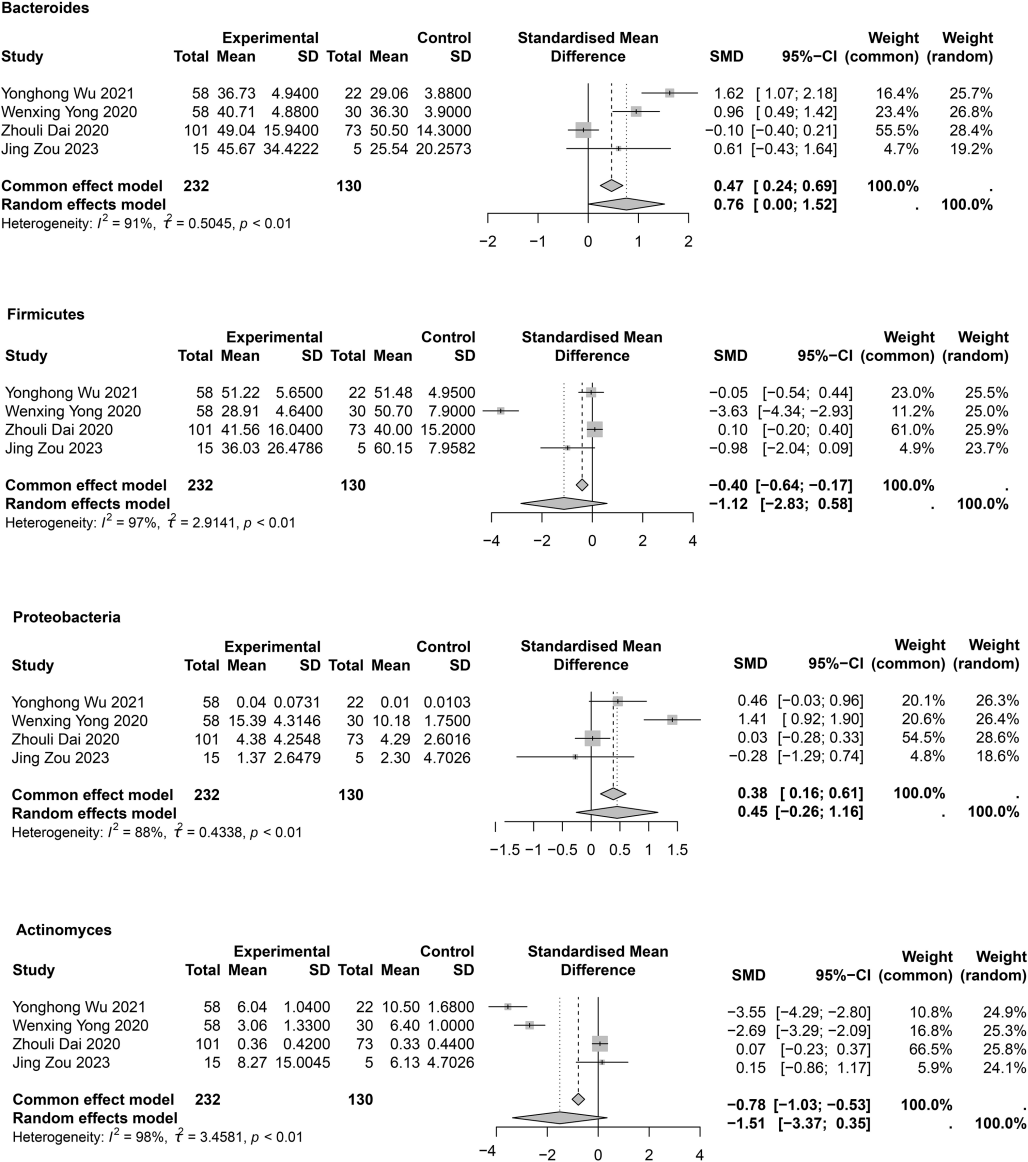
Meta-analysis of phylum level in COPD patients.

At the phylum level, mainly Bacteroidetes, Proteobacteria, Firmicutes, and Actinomycetota were studied (**Supplementary Table 5**, **Figure 4**). In the meta-analysis for Bacteroidetes (**Figure 4**), a statistical difference was observed between groups (SMD = 0.76; 95% CI, 0.00 to 1.52; *I* ^2^ = 91%; τ^2^ = 0.5045). Sensitivity analysis showed that after removal of Zhouli Dai’ study (Dai, 2020), heterogeneity was reduced to I² = 56% (**Supplementary Figure 1**), and the meta-analysis results were as follows: SMD = 1.14; 95% CI, 0.59 to 1.69. This result may be due to the inclusion of patients with varying degrees of COPD among the enrolled patients. In four studies (Dai, 2020; Yong et al., 2020; Wu et al., 2021; Zou et al., 2023). Proteobacteria were abundant in patients with COPD; however, no statistical difference between groups was observed (SMD = 0.45; 95% CI, −0.26 to 1.16; *I* ^2^ = 88%; τ^2^ = 0.4338) (**Figure 4**). Sensitivity analysis showed that after removal of Wenxing Yong’s study (Yong et al., 2020), heterogeneity decreased to 29% (**Supplementary Figure 1**), but the meta-analysis still showed no difference between groups (SMD = 0.14; 95% CI, −0.21 to 0.48; *I* ^2^ = 29%; τ^2^ = 0.0309). Four studies (Dai, 2020; Yong et al., 2020; Wu et al., 2021; Zou et al., 2023) provided meta-analyses of Firmicutes (SMD = -1.12; 95% CI, -2.83 to 0.58; *I* ^2^ = 97%; τ^2^ = 2.9141) and Actinomycetota (SMD = −1.51; 95% CI, −3.37 to 0.35; *I* ^2^ = 98%; τ^2^ = 3.4581). No statistical differences were observed between COPD and HC groups (**Figure 4**). Owing to large heterogeneity, the quality of evidence for these outcomes was rated as low. Sensitivity analyses showed no differences between groups; however, heterogeneity was significantly reduced (**Supplementary Figure 1**). Publication bias assessments are presented in **Supplementary Table 6** and indicated no significant publication bias.

For asthma-associated changes in gut flora at the phylum level, see **Supplementary Table 5**. Two studies (Ishaq et al., 2018; Ming et al., 2024) showed an increase in Bacteroidetes in patients with asthma compared to HCs, while Zou et al. (2021) found the opposite. No consistent pattern of change was observed at the phylum level in patients with asthma.

### Genus

**Figure 5:**
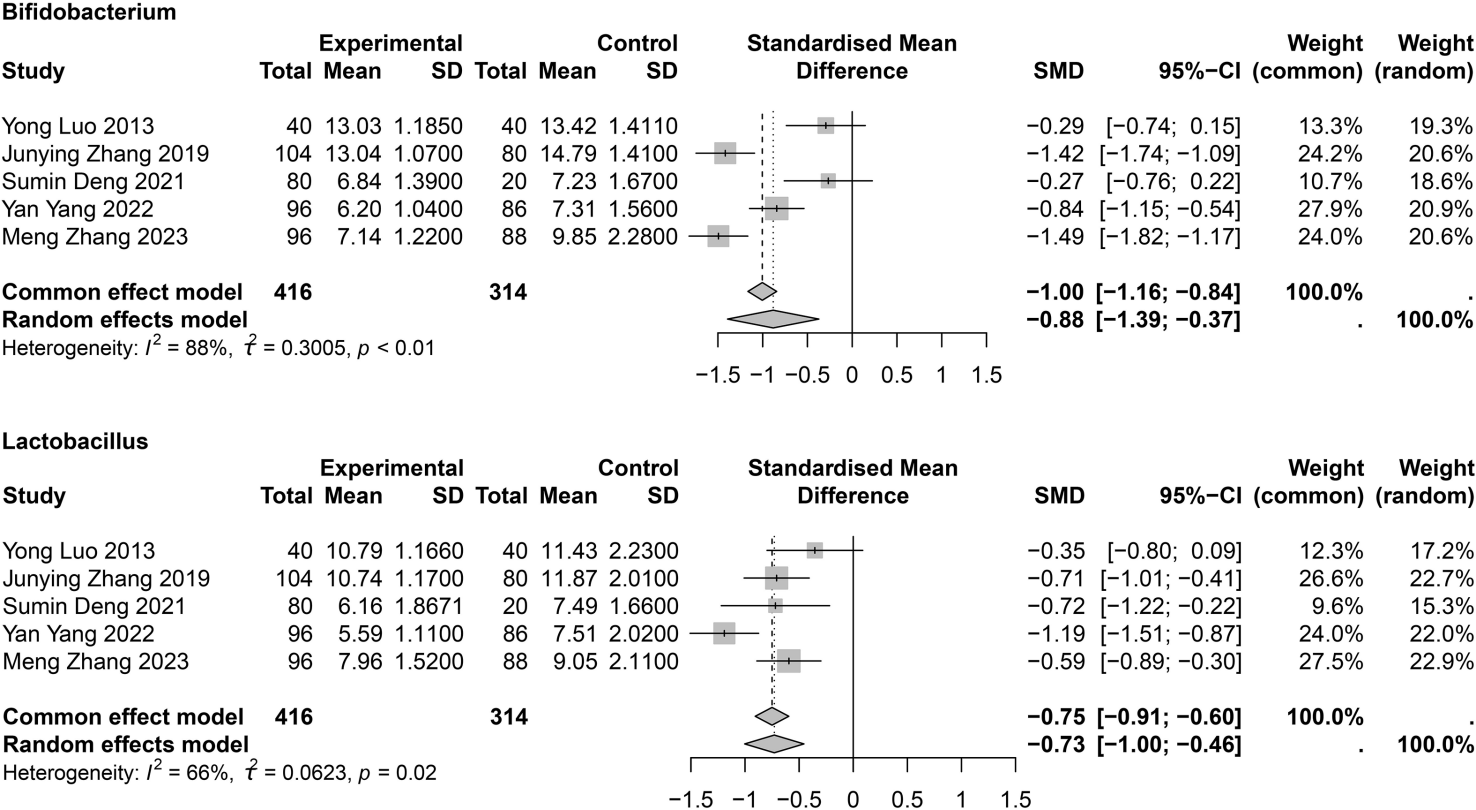
Meta-analysis of *Bifidobacterium* and *Lactobacillus* in COPD patients.

For variability of flora between COPD and HC groups, three studies showed lower Bacteroides levels in patients with COPD (Wu et al., 2021; Zhang, 2021; Jiao Qianqian et al., 2022), whereas two studies (Bowerman et al., 2020; Chen, 2021) reported the opposite. Two studies reported that *Enterococcus faecalis, Enterococcus faecium* (Deng, 2021; Yang et al., 2022) and *Rothia (* Bowerman et al., 2020; Yong et al., 2020) levels were higher in patients with COPD than in HCs. Six studies (Luo et al., 2013; Junying, 2019; Yong et al., 2020; Deng, 2021; Wu et al., 2021; Yang et al., 2022) investigated *Bifidobacterium*, and five studies (Luo et al., 2013; Junying, 2019; Deng, 2021; Yang et al., 2022; Zhang et al., 2023) showed that *Lactobacillus* levels were lower in patients with COPD (**Supplementary Table 5**). The meta-analysis indicated that *Bifidobacterium* (SMD = −0.88; 95% CI, −1.39 to −0.37; *I* ^2^ = 88%; τ^2^ = 0.3005) and *Lactobacillus* (SMD = −0.73; 95% CI, −1.00 to −0.46; *I* ^2^ = 66%; τ^2^ = 0.0623) levels were significantly lower in patients with COPD than in HCs (**Figure 5**). Sensitivity analysis showed that the differences persisted; however, heterogeneity was significantly reduced (**Supplementary Figure 1**). Other genus-level differences are detailed in **Supplementary Table 5**, showing that beneficial intestinal flora were reduced in patients with COPD compared to HCs. Crude values of the different indicators in patients with COPD or asthma are shown in **Supplementary Table 7**.

## Discussion

In this first meta-analysis to systematically assess gut microbiota dysbiosis in patients with COPD and asthma, we found that the α-diversity of intestinal flora in patients with asthma did not differ from that of healthy individuals; however, the α-diversity of the intestinal flora in patients with COPD differed from that of HCs. Moreover, the *Bifidobacterium* and *Lactobacillus* levels decreased, suggesting the possibility of gut microbiota dysbiosis in patients with COPD and providing a potential strategy for the clinical management and treatment of COPD.

Trillions of microorganisms, with up to 1,000 species, reside in the gastrointestinal tract (Krumina et al., 2022). The proportion of anaerobic bacteria in normal intestinal flora is significantly higher than that of aerobic bacteria. *Bacteroides*, *Bifidobacterium*, and *Eubacillus* are among the anaerobic bacteria that are dominant, whereas *Escherichia*, *Enterobacter*, and *Enterococcus* are dominant among the aerobic bacteria. The gut microbiota has important and specific metabolic, nutritional, and protective functions (Guarner and Malagelada, 2003). The number of intestinal fungi and lactose-fermenting bacteria (*Streptococcus*, *Lactobacillus*, and *Klebsiella*) is increased in patients with inflammatory bowel disease (Lewis et al., 2015). In the inflamed mucosa of patients with colitis, gram-negative Proteobacteria are increased, while gram-positive Firmicutes are decreased (Xu et al., 2018). Butyrate-producing Bacillus faecalis and Coccus faecalis have been linked to a higher quality of life and better mental health (Valles-Colomer et al., 2019). In addition, several diseases such as diabetes mellitus, obesity, autoimmune diseases, and cancer occur with gut microbiome dysbiosis, showing that the microbiome plays a crucial role in the body.

Moreover, there appears to be a consensus that reduced α-diversity of the gut microbiota is harmful to the host-organism (Ren et al., 2019; Agus et al., 2021). Modifications in bacterial homeostasis are initiated when the richness and evenness of the intestinal flora are disrupted. As reported by Lim et al (Lim et al., 2023), the configuration of intestinal bacteria, metabolites, and other elements exerts a considerable influence on conditions such as COPD and asthma. The levels of intestinal flora in patients with COPD were negatively correlated with IL-6, IL-8, and TNF-α levels and positively correlated with pulmonary function indicators (Yang et al., 2022). Furthermore, the transplantation of feces from patients with COPD into mice resulted in a notable elevation in IL-1 and TNF-α levels, accompanied by the induction of lung inflammation, compared to the control group (Dai, 2020). In our study, we found that Shannon’s index was reduced in patients with COPD according to the meta-analysis and that α-diversity may be a discriminating indicator for COPD. Regarding β-diversity, there were significant differences in the composition of the gut flora between patients with COPD and HCs. In conclusion, the above findings reveal the possibility of an altered intestinal flora profile in patients with COPD.

Subsequently, further investigations were conducted to ascertain whether alterations had occurred at the phylum level within the gut microbiota. The results of the meta-analysis indicated that the Bacteroidetes population increased in patients with COPD compared to HCs. Four studies (Yong et al., 2020; Wu et al., 2021; Zhang, 2021; Zou et al., 2023) reported reduced Firmicutes levels in patients with COPD compared to HCs, and two studies (Wu et al., 2021; Zhang, 2021) found elevated Proteobacteria levels in patients with COPD. Lee et al. found reduced Firmicutes and Proteobacteria levels in patients with COPD compared to nonsmokers (Lee et al., 2018), and that smoking was the main cause of COPD. However, Sun et al. found an increase in the relative abundance of Proteobacteria and a decrease in Firmicutes in patients with worsening COPD (Sun et al., 2020), and a positive correlation between Proteobacteria and elevated IL-6 and IL-8 levels (Samuelson et al., 2015). These inflammatory factors play an important role in the chronic inflammation of COPD. Firmicutes contain most butyrate-producing microorganisms (Singhal et al., 2021); butyrate has an important role in intestinal barrier integrity and intestinal homeostasis, while also increasing the antimicrobial capacity of macrophages (Schulthess et al., 2019), suggesting that a reduction in Firmicutes in patients with COPD is detrimental to health. Although the meta-analysis showed no statistical difference in Firmicutes and Proteobacteria levels between patients with COPD and healthy individuals, the large heterogeneity, as well as the presence of selection bias, make this finding less interpretable. Moreover, the evidence in this regard is limited and requires further validation in more studies.

At the genus level, *Bifidobacterium* and *Lactobacillus* were found to be reduced in COPD according to the meta-analysis, and these two microorganisms are classified as probiotics. Probiotics are defined as “living microorganisms that, when consumed in sufficient amounts, confer health benefits on the host,” the most common of which are the lactic acid–producing *Bifidobacterium* and *Lactobacillus* genera present in the gut (Hills et al., 2019). Similarly, prebiotics, which have a similar effect, are indigestible food components that benefit the host by selectively stimulating the growth or activity of one or a limited number of bacteria in the colon. Prebiotics include FOS, GOS, and polyol sugar alcohols, which are used as nutritional sweeteners. Studies have reported a significant reduction in the duration of diarrhea in children with acute gastroenteritis treated with probiotics (Guandalini et al., 2000), a positive effect in the treatment of type 2 diabetes mellitus, and improved depression-related behavior in rats (Abildgaard et al., 2017; Sabico et al., 2019). Furthermore, in mouse models, oral administration of probiotics reduced the number of inflammatory cells and lung Th2 cytokines and decreased local inflammatory responses (Wu et al., 2016). Probiotics also helped to maintain the stability of intestinal flora diversity and may prevent and improve allergies and respiratory diseases (Ozdemir, 2010; Liu et al., 2021). The level of inflammation in patients with COPD was significantly lower after administration of probiotics than before treatment, and improved the clinical symptoms of patients (Deng, 2021). These results warrant consideration of probiotics in the treatment of COPD. Probiotic treatment yielded favorable outcomes in mice with COPD, indicating its potential therapeutic efficacy. Probiotics reinforce the intestinal epithelial barrier by tightening intercellular junctions and sealing tight junctions, thereby curbing bacterial translocation (Yuksel et al., 2023). Concurrently, they stimulate the production of antimicrobial peptides and secretory IgA to exert direct bacteriostatic effects (Budden et al., 2017). On the immunoregulatory front, probiotics skew the immune response toward an anti-inflammatory profile: they expand CD4^+^Foxp3^+^ regulatory T cells, dampen pro-inflammatory cytokine release, and drive T-cell polarization toward a Th1 phenotype (Kwon et al., 2010). Moreover, a decline in Bifidobacteria and Bacteroidaceae diminishes short-chain fatty acid (SCFA) synthesis (Wang et al., 2021). SCFAs, in turn, safeguard pulmonary function both directly—by preserving lung immune homeostasis—and indirectly—by maintaining intestinal barrier integrity (Song et al., 2024). Specifically, butyrate attenuates Th9-mediated immunity to mitigate pulmonary inflammation, whereas propionate and butyrate curb histone deacetylase activity and foster Treg differentiation, alleviating inflammation in patients with COPD (DeMarini, 2004; de Souza Vieira et al., 2019). As the primary energy source for colonic epithelial cells, SCFAs further strengthen the gut barrier, effectively preventing luminal dissemination of pathogens and endotoxins (Trompette et al., 2014). However, further validation in large-scale randomized controlled trials is essential for clinical application.

COPD is a chronic, irreversible, and progressive disease that is a leading cause of mortality and morbidity worldwide. The findings of this study indicate that patients with COPD exhibit a reduction in intestinal probiotics. Additionally, the use of glucocorticosteroids and antimicrobial drugs disrupts intestinal homeostasis. Probiotics are beneficial flora of the human intestine that can inhibit the growth of pathogenic microorganisms, increase antibody levels, and promote macrophage activity to enhance immune function. It can be reasonably proposed, therefore, that probiotic supplementation therapy should be introduced for patients with COPD.

In our study, most of the included literature originated from China. This may be related to data from the 2019 Burden of Disease in China report, which indicated that COPD has become the third leading cause of death in China (Zhou et al., 2019). Furthermore, the acceleration of population aging in China is accompanied by an increase in the health burden of chronic non-communicable diseases, which are closely related to age in the older adult population. By the end of 2018, the population aged ≥60 years in China had reached 249 million, representing approximately 20% of the total population. It is projected that by 2050, the older adult population will exceed 400 million, with an aging proportion exceeding 30% (Yu, 2019). As COPD predominantly affects older adults, the 2015 Global Burden of Disease Study indicated that China had the second-highest age-standardized disability-adjusted life-year rate worldwide due to COPD (GBD 2015 Chronic Respiratory Disease Collaborators, 2017). COPD represents a significant threat to the health of the Chinese population and constitutes a formidable challenge to be addressed. Furthermore, it should be noted that the literature selection in this study may have been subject to some degree of bias.

The study had some limitations. First, the sample size of the included studies was relatively small, which may be owing to the literature selection methodology and may lead to uncertainty and reduced accuracy of the results. Second, as the number of studies for the meta-analysis was fewer than seven, the results may require further validation. Further evidence is needed to substantiate the relationship between the incidence of COPD/CLD and gut microbiota. Third, owing to limited data, we did not perform subgroup analyses based on the source of specimens, disease severity, frequency of exacerbations, presence of comorbidities, or treatment with corticosteroids. These factors may influence the composition of the gut flora and therefore require further exploration. Fourth, as all the studies were conducted in China, screening bias cannot be ruled out. Differences in gut flora among patients with COPD according to ethnicity could not be determined.

## Conclusion

The gut microbiota of patients with COPD is imbalanced, and the abundance of probiotics is lower than in healthy individuals. Further exploration of the potential mechanisms of probiotics in patients with COPD may provide promising targets for treatment. However, the heterogeneity and limited number of included studies should be considered. Nevertheless, systematic exploration of this mechanism is expected to provide a promising breakthrough for individualized treatment and management of COPD.

## Data Availability

The original contributions presented in the study are included in the article/**Supplementary Material**. Further inquiries can be directed to the corresponding authors.
