# Supplementary material for: Gut microbiome dysbiosis in chronic lung disease: a systematic review and meta-analysis
Source: Front Cell Infect Microbiol. 2025 Oct 10;15:1554846. doi: 10.3389/fcimb.2025.1554846 (PMC12549607; doi:10.3389/fcimb.2025.1554846)

Supplementary Material

**Supplementary Table 1.** Systematic search detail.

**Supplementary Table 2.** The characteristics of included studies.

**Supplementary Table 3.** Assessment of risk of bias in non-interventional comparative included studies.

**Supplementary Table 4.** Beta diversity between CLD and HC.

**Supplementary Table 5.** Differentially abundant microbial taxa.

**Supplementary Table 6.** Egger test between CLD and HC.

**Supplementary Table 7.** The crude values of the different indicators in the COPD or asthma patients.

**Supplementary Figure 1.** Sensitivity analysis.

**Supplementary Figure 2.** Subgroup analysis of chao1 in the COPD patients according to the traditional Chinese medicine treatment.

**Supplementary Table 1. Systematic search detail.**

- 1. **Pubmed**

| **Sequence** | **Search** | **Hits** |
| --- | --- | --- |
| 1 | (((((((((((((((((((((asthma[Title/Abstract]) OR (Asthma[MeSH Terms])) OR (chronic obstructive pulmonary disease[Title/Abstract])) OR (COPD[Title/Abstract])) OR (emphysema[Title/Abstract])) OR (chronic bronchitis[Title/Abstract])) OR (bronchiectasis[Title/Abstract])) OR ("Hypertension, Pulmonary"[MeSH Terms])) OR (cystic fibrosis[Title/Abstract])) OR (Cystic Fibrosis[MeSH Terms])) OR (pulmonary arterial hypertension[Title/Abstract])) OR (Pulmonary Disease, Chronic Obstructive[MeSH Terms])) OR (airway obstruction[Title/Abstract])) OR (bronchoconstriction[Title/Abstract])) OR (expiratory airflow limitation[Title/Abstract])) OR (lung disease[Title/Abstract])) OR (bronchopulmonary disease[Title/Abstract])) OR (pulmonary disease[Title/Abstract])) OR (airways disease[Title/Abstract])) OR (idiopathic pulmonary fibrosis[Title/Abstract])) OR (chronic respiratory disease[Title/Abstract])) OR (chronic lung disease[Title/Abstract]) | 498959 |
| 2 | (((((gut[Title/Abstract]) OR (gastrointestinal[Title/Abstract])) OR (intestinal[Title/Abstract])) OR (feacal[Title/Abstract])) OR (fecal[Title/Abstract])) OR (stool[Title/Abstract]) | 795226 |
| 3 | ((((((microbiome[Title/Abstract]) OR (microbiota[Title/Abstract])) OR (ecosystem[Title/Abstract])) OR (bacteria[Title/Abstract])) OR (flora[Title/Abstract])) OR (microflora[Title/Abstract])) OR (dysbiosis[Title/Abstract]) | 716150 |
| 4 | 1 AND 2 AND 3 | 1769 |

**1.2 Web of science**

| **Sequence** | **Search** | **Hits** |
| --- | --- | --- |
| 1 | (AB=asthma OR Asthma OR chronic obstructive pulmonary disease OR COPD OR emphysema OR chronic bronchitis OR bronchiectasis OR "Hypertension, Pulmonary" OR cystic fibrosis OR Cystic Fibrosis OR pulmonary arterial hypertension OR Pulmonary Disease, Chronic Obstructive OR airway obstruction OR bronchoconstriction OR expiratory airflow limitation OR lung disease OR bronchopulmonary disease OR pulmonary disease OR airways disease OR idiopathic pulmonary fibrosis OR chronic respiratory disease OR chronic lung disease) | 715196 |
| 2 | (AB=gut OR gastrointestinal OR intestinal OR feacal OR fecal OR stool) | 896190 |
| 3 | (AB=microbiome OR microbiota OR ecosystem OR bacteria OR flora OR microflora OR dysbiosis) | 1311146 |
| 4 | 1 AND 2 AND 3 | 3389 |

**1.3 Cochrane**

| **Sequence** | **Search** | **Hits** |
| --- | --- | --- |
| 1 | (asthma):ab,ti,kw OR (Asthma):ab,ti,kw OR (chronic obstructive pulmonary disease):ab,ti,kw OR (COPD):ab,ti,kw OR (emphysema):ab,ti,kw OR (chronic bronchitis):ab,ti,kw OR (bronchiectasis):ab,ti,kw OR ("Hypertension, Pulmonary"):ab,ti,kw OR (cystic fibrosis):ab,ti,kw OR (Cystic Fibrosis):ab,ti,kw OR (pulmonary arterial hypertension):ab,ti,kw OR (Pulmonary Disease, Chronic Obstructive):ab,ti,kw OR (airway obstruction):ab,ti,kw OR (bronchoconstriction):ab,ti,kw OR (expiratory airflow limitation):ab,ti,kw OR (lung disease):ab,ti,kw OR (bronchopulmonary disease):ab,ti,kw OR (pulmonary disease):ab,ti,kw OR (airways disease):ab,ti,kw OR (idiopathic pulmonary fibrosis):ab,ti,kw OR (chronic respiratory disease):ab,ti,kw OR (chronic lung disease):ab,ti,kw | 108346 |
| 2 | (gut):ab,ti,kw OR (gastrointestinal):ab,ti,kw OR (intestinal):ab,ti,kw OR (feacal):ab,ti,kw OR (fecal):ab,ti,kw OR (stool):ab,ti,kw | 89299 |
| 3 | (microbiome):ab,ti,kw OR (microbiota):ab,ti,kw OR (ecosystem):ab,ti,kw OR (bacteria):ab,ti,kw OR (flora):ab,ti,kw OR (microflora):ab,ti,kw OR (dysbiosis):ab,ti,kw | 29524 |
| 4 | 1 AND 2 AND 3 | 0 |

**1.4 Embase**

| **Sequence** | **Search** | **Hits** |
| --- | --- | --- |
| 1 | 'asthma':ab,ti OR 'Asthma':ab,ti OR 'chronic obstructive pulmonary disease':ab,ti OR 'COPD':ab,ti OR 'emphysema':ab,ti OR 'chronic bronchitis':ab,ti OR 'bronchiectasis':ab,ti OR 'Hypertension, Pulmonary':ab,ti OR 'cystic fibrosis':ab,ti OR 'Cystic Fibrosis':ab,ti OR' pulmonary arterial hypertension':ab,ti OR 'Pulmonary Disease, Chronic Obstructive':ab,ti OR 'airway obstruction':ab,ti OR 'bronchoconstriction':ab,ti OR 'expiratory airflow limitation':ab,ti OR 'lung disease':ab,ti OR 'bronchopulmonary disease':ab,ti OR 'pulmonary disease':ab,ti OR 'airways disease':ab,ti OR 'idiopathic pulmonary fibrosis':ab,ti OR 'chronic respiratory disease':ab,ti OR 'chronic lung disease':ab,ti | 663819 |
| 2 | 'gut':ab,ti OR 'gastrointestinal':ab,ti OR' intestinal':ab,ti OR 'feacal':ab,ti OR 'fecal':ab,ti OR 'stool':ab,ti | 1053147 |
| 3 | 'microbiome':ab,ti OR 'microbiota':ab,ti OR 'ecosystem':ab,ti OR 'bacteria':ab,ti OR 'flora':ab,ti OR 'microflora':ab,ti OR 'dysbiosis':ab,ti | 798010 |
| 4 | 1 AND 2 AND 3 | 2589 |

**1.5 CNKI**

| **Sequence** | **Search** | **Hits** |
| --- | --- | --- |
| 1 | SU%=(asthma + Asthma + chronic obstructive pulmonary disease + COPD + emphysema + chronic bronchitis + bronchiectasis + Hypertension, Pulmonary + cystic fibrosis + Cystic Fibrosis + pulmonary arterial hypertension + Pulmonary Disease, Chronic Obstructive + airway obstruction + bronchoconstriction + expiratory airflow limitation + lung disease + bronchopulmonary disease + pulmonary disease + airways disease + idiopathic pulmonary fibrosis + chronic respiratory disease + chronic lung disease) | 391726 |
| 2 | SU%=(gut + gastrointestinal + intestinal + feacal + fecal + stool) | 186243 |
| 3 | SU%=(microbiome + microbiota + ecosystem + bacteria + flora + microflora + dysbiosis) | 815943 |
| 4 | 1 AND 2 AND 3 | 427 |

**1.6 Wanfang**

| **Sequence** | **Search** | **Hits** |
| --- | --- | --- |
| 1 | Title or keywords: (Chronic Obstructive Lung Disease OR Chronic Obstructive Pulmonary Diseases OR COAD OR COPD OR Chronic Obstructive Airway Disease OR Chronic Obstructive Pulmonary Disease OR Airflow Obstruction, Chronic OR Airflow Obstructions, Chronic OR Chronic Airflow Obstructions) | 112 |
| 2 | Title or keywords: (gut OR gastrointestinal OR intestinal OR feacal OR fecal OR stool) | 112 |
| 3 | Title or keywords: (microbiome OR microbiota OR ecosystem OR bacteria OR flora OR microflora OR dysbiosis) | 112 |
| 4 | 1 AND 2 AND 3 | 112 |

**1.7 VIPC**

| **Sequence** | **Search** | **Hits** |
| --- | --- | --- |
| 1 | R =(asthma OR Asthma OR chronic obstructive pulmonary disease OR COPD OR emphysema OR chronic bronchitis OR bronchiectasis OR Hypertension, Pulmonary OR cystic fibrosis OR Cystic Fibrosis OR pulmonary arterial hypertension OR Pulmonary Disease, Chronic Obstructive OR airway obstruction OR bronchoconstriction OR expiratory airflow limitation OR lung disease OR bronchopulmonary disease OR pulmonary disease OR airways disease OR idiopathic pulmonary fibrosis OR chronic respiratory disease OR chronic lung disease) | 382111 |
| 2 | R = (gut OR gastrointestinal OR intestinal OR feacal OR fecal OR stool) | 61491 |
| 3 | R = (microbiome OR microbiota OR ecosystem OR bacteria OR flora OR microflora OR dysbiosis) | 274595 |
| 4 | 1 AND 2 AND 3 | 411 |

**Supplementary Table 2. The characteristics of included studies.**

| **Study** | **Country** | **Disease** | **CLD (n, male/female)** | **Control, (n, male/female)** | **CLD (age)** | **Control (age)** | **Sample source and storage** | **Analysis methods** | **Level** | **Design** |
| --- | --- | --- | --- | --- | --- | --- | --- | --- | --- | --- |
| Chiyong Chen et al 2023(45) | China | COPD | 80 (NA) | 80 (NA) | 62.9479±5.1491 | 62.48±5.75 | Fecal, -80°C | Metagenomic sequencing | NA | Case-control |
| Xiaoli Chen et al 2021(38) | China | COPD | 59 (42/17) | 30 (18/12) | 71.4585±10.5711 | 68.57±8.00 | Fecal, -20°C | 16S rRNA | OTU | Case-control |
| Yuexuan Chen et al 2018(34) | China | COPD | 58 (27/31) | 30 (15/15) | 63.1886±6.1609 | 64.11±5.29 | Fecal, -80°C | 16S rRNA | OTU | Case-control |
| Meng Zhang et al 2023(57) | China | COPD | 96 (60/36) | 88 (50/38) | 60.36±2.28 | 60.85±2.55 | Fecal, NA | bacterial culture | NA | Case-control |
| Zhouli Dai et al 2020(39) | China | COPD | 101 (NA) | 73 (NA) | 62.3±6.7 | 59.1±9.4 | Fecal, -80°C | 16S rRNA | OTU | Case-control |
| Sumin Deng et al 2021(54) | China | COPD | 80 (44/36) | 20 (10/10) | 72.5±10.0 | 64.9±9.4 | Fecal, -80°C | bacterial culture | NA | Case-control |
| Yonghong Wu et al 2021(32) | China | COPD | 29 (18/11) | 22 (10/12) | 65.93±8.3 | 46.55±13.8 | Fecal, NA | 16S rRNA | OTU | Case-control |
| Yuting Kang et al 2022(43) | China | COPD | 10 (8/2) | 10 (7/3) | 65.3±7.7 | 63.1±8.6 | Fecal, -80°C | Metagenomic sequencing | NA | Case-control |
| Qianqian Jiao et al 2022(40) | China | COPD | 22 (13/9) | 21 (11/10) | 68.4±7.9 | 54.1±12.9 | Fecal, -80°C | 16S rRNA | OTU | Case-control |
| Yong Luo et al 2013(55) | China | COPD | 40 (34/6) | 40 (34/7) | 64.0±5.0 | 63.0±7.0 | Fecal, -80°C | 16S rRNA | OTU | Case-control |
| Yukun Ma et al 2019(86) | China | COPD | 70 (32/38) | 30, NA | NA | 72.1±10.5 | Fecal, -80°C | 16S rRNA | OTU | Case-control |
| Junying Zhang et al 2019(56) | China | COPD | 104 (59/45) | 80 (32/48) | 34.9±18.9 | 46.9±18.9 | Fecal, -80°C | 16SrRNA/DNA | OTU | Case-control |
| Yu zhang et al 2021(41) | China | COPD | 11 (NA) | 9 (NA) | 72.5±10.0 | 68.1±4.7 | Fecal, 4°C | 16S rRNA | OTU | Case-control |
| Yimeng Xu et al 2022(51) | China | COPD | 10 (NA) | 10 (NA) | 61.20±7.07 | 57.80±9.4 | Fecal, -80°C | Metagenomic next generation sequencing | NA | Case-control |
| Jing Zou et al 2023(44) | China | COPD | 15 (13/2) | 5 (33/2) | 71.726±8.18 | 69.5541±8.10 | Fecal, -80°C | Metagenomics sequencing | NA | Case-control |
| Yang Liu et al 2022(42) | Finland | COPD | 145 (102/43) | 5932 (2728/3204) | 59.9±9.7 | 49.7±15.1 | Fecal, -20°C | shallow shotgun metagenomic sequencing | GTDB | Cohort study |
| Jiali Yan et al 2024(52) | China | COPD | 60 (NA) | 10 (NA) | 72.2±7.4455 | NA | Fecal, -80°C | 16S rRNA | OTU | Case-control |
| Naijian Li et al 2021(20) | China | COPD | 99 (NA) | 73 (NA) | 61.9± 6.62 | 59.7±9.4 | Fecal, -80°C | 16S rRNA | OTU | Case-control |
| Wenxing  Yong et al 2020(33) | China | COPD | 60 (27/33) | 30 (15/15) | NA | NA | Fecal, -80°C | 16S rRNA | OTU | Case-control |
| Yan Yang et  al 2022(53) | China | COPD | 96 (69/27) | 96 (64/22) | 62.4±13.2 | 61.0±11.9 | Fecal, -80°C | bacterial culture | NA | Case-control |
| Kate L. Bowerman et al 2020(50) | Australian | COPD | 28 (15/13) | 29 (19/10) | 67.0±7.2 | 60.4±11.6 | Fecal, -80°C | 16S rRNA | OTU | Case-control |
| Yaoxin Chen et al 2023(48) | China | asthma | 28 (5/23) | 11 (3/11) | 58.25±1.24 | 54.45±3.56 | Fecal, -80°C | 16S rRNA | OTU | Case-control |
| Ming Liu et al 2024(47) | China | asthma | 14 (8/6) | 15 (8/7) | 47.38±9.04 | 46.92±8.98 | Fecal, -80°C | NA | NA | Case-control |
| Hafiz Muhammad Ishaq et al 2018(46) | China | asthma | 15 (7/8) | 5 (2/3) | NA | NA | Fecal, -80°C | NA | NA | Case-control |
| Yang Liu et al 2022(42) | China | asthma | 435 (183/252) | 5244 (2504/2740) | 50.6±14.88 | 49.6±14.90 | Fecal, -20°C | shallow shotgun metagenomic sequencing | GTDB | Cohort study |
| О.Yu. Zolnikova et al 2020(87) | Russia | asthma | 20 (11/9) | 15 (6/9) | 43.6±12.5 | 46.7±9.7 | Fecal, -20°C | NA | NA | Cohort study |
| Xiaoling Zou et al 2021(49) | China | asthma | 47 (22/25) | 20 (10/10) | 39.91±16.02 | 20.64±14.53 | Fecal, -80°C | 16S rRNA | OTU | Case-control |
| D. G. Burke, F et al 2017(88) |  | CF | 43 (25/18) | 69 (49/20) | 29 ± 8.3 | 32±8.1 | Fecal, -80°C | 16S rRNA | OTU | Case-control |

Note: GTDB, Genome Taxonomy Database, a project aimed at establishing a standardized classification of microorganisms based on genomic phylogenetics; OUT, Operational Taxonomic Unit, mainly used to describe bacterial communities by amplicon sequencing; NA: not acquire.

Reference

20. Li N, Dai Z, Wang Z, Deng Z, Zhang J, Pu J, Cao W, Pan T, Zhou Y, Yang Z, Li J, Li B, Ran P. Gut microbiota dysbiosis contributes to the development of chronic obstructive pulmonary disease. Respir Res. 2021;22(1):274. eng. 4.7. doi:10.1186/s12931-021-01872-z. Cited in: Pubmed; PMID 34696775.

32. Wu Y, Luo Z, Liu C. Variations in fecal microbial profiles of acute exacerbations and stable chronic obstructive pulmonary disease. Life Sci. 2021 Jan 15;265:118738. eng. Epub 20201110. doi:10.1016/j.lfs.2020.118738. Cited in: Pubmed; PMID 33181175.

33. Yong W, Zhang L, Chen Y, Li J, Liu Y, Zhang Z. Jianpi Huatan Tongfu granule alleviates inflammation and improves intestinal flora in patients with acute exacerbation of chronic obstructive pulmonary disease. J Int Med Res. 2020 Apr;48(4):300060520909235. eng. doi:10.1177/0300060520909235. Cited in: Pubmed; PMID 32295442.

34. Chen Y. The therapeutic mechanism of phlegm antiasth matic method in the treatment of AECOPD based on the analysis of intestinal microecological changes based on “Lung and Large Intestine Have Relevance”: Gansu University of Chinese Medicine; 2018.

38. Chen X. Based on "the lungs and the large intestine are the same between the outside and the inside", the mechanism of the therapeutic effect of Qingjin Huatan Decoction on AECOPD is analyzed by studying the changes of intestinal flora. [master]: Guangxi University of Chinese Medicine; 2021. Cnki.

39. Dai Z. The role of gut-lung axis in chronic obstructive pulmonary disease and its mechanism [master]: Guangzhou Medical University; 2020. Cnki.

40. Jiao Qianqian YJ, Gao Fuquan, Zhai Nailiang, Yuan Fei, Lu Feng, Pan Lei. . Comparison of the difference of gut microbiota between patients with chronic obstructive pulmonary disease and helthy controls. BMU Journal. 2022;45(03):177-181. doi:10.19739/j.cnki.issn1001-9510.2022.03.004.

41. Zhang Y. Study on Microecological Status of Respiratory Tract Flora and Intestinal Flora in Patients with Acute Exacerbation of Chronic Obstructive Pulmonary Disease [master]: Dalian Medical University; 2021. Cnki.

42. Liu Y, Teo SM, Meric G, Tang HHF, Zhu Q, Sanders JG, Vazquez-Baeza Y, Verspoor K, Vartiainen VA, Jousilahti P, Lahti L, Niiranen T, Havulinna AS, Knight R, Salomaa V, Inouye M. The gut microbiome is a significant risk factor for future chronic lung disease. 2022;preprint. doi:10.1101/2022.03.22.22272736.

43. KANG Yu-ting, SU Ya-jing, QIAO Xia, WANG Peng-tao, YANG Ning-ai, ZHAO Zhi-jun, JIA Wei. Gut microbial composition and gene function in patients with chronic obstructive pulmonary disease. Chinese Journal of Microecology. 2022;34(10):1122-1128. doi:10.13381/j.cnki.cjm.202210002.

44. Zou J, Xu X, Chen Y, Sha W, Qin H. Association between gut microbiota and clinical features in patients with acute exacerbation chronic obstructive pulmonary disease based on metagenomics study. Journal of Tongji University (medical science). 2023;44(01):41-51. doi:10. 12289/j. issn. 1008-0392. 22189.

45. Chen C. Changes in intestinal flora composition and its effects in chronic obstructive pulmonary disease [master]: Guangzhou Medical University; 2023.

46. Ishaq HM, Shahzad M, Wu X, Ma C, Xu J. Gut Microbe Analysis between Asthma Patients and Healthy Volunteers in Shaanxi Province, Xian, China. Pakistan Journal of Zoology. 2018;50(1). doi:10.17582/journal.pjz/2018.50.1.165.173.

47. Ming L, Jie G, Dan Z, Xuan Z. Characteristics of intestinal flora in patients with allergic asthma. Chinese Journal of Respiratory and Critical Care. 2024;23(01):1-6. doi:10.7507/1671-6205.202304057.

48. Chen Y. Characterization of Gut Microbiota in Patients with Different Airflow Obstruction Phenotypes of Asthma: Traditional Chinese Medicine University Of Guangzhou; 2023.

49. Zou X-L, Wu J-J, Ye H-X, Feng D-Y, Meng P, Yang H-L, Wu W-B, Li H-T, He Z, Zhang T-T. Associations Between Gut Microbiota and Asthma Endotypes: A Cross-Sectional Study in South China Based on Patients with Newly Diagnosed Asthma. Journal of Asthma and Allergy. 2021;Volume 14:981-992. doi:10.2147/jaa.S320088.

50. Bowerman KL, Rehman SF, Vaughan A, Lachner N, Budden KF, Kim RY, Wood DLA, Gellatly SL, Shukla SD, Wood LG, Yang IA, Wark PA, Hugenholtz P, Hansbro PM. Disease-associated gut microbiome and metabolome changes in patients with chronic obstructive pulmonary disease. Nat Commun. 2020 Nov 18;11(1):5886. eng. P.H. is a co-founder of Microba Life Sciences Limited, and D.L.A.W. is currently an employee of Microba. The remaining authors declare no competing interests. Epub 20201118. doi:10.1038/s41467-020-19701-0. Cited in: Pubmed; PMID 33208745.

51. Yimeng X, Hao W, Difei L, Taobin C, Guansheng S, Peiyan Z, Huiyin X, Shixian Y, Lulu W, Ni L, Weiwei S, Zeguang Z. Comparison of gut microflora between stable chronic obstructive pulmonary disease patients and

healthy controls by metagenomic second generation sequencing. Int J Respir. 2022;42(18):1361-1369. doi:10.3760/cma.j.cn131368-20220511-00377.

52. Yan J, Wu Z, Deng L, Huang C, Jing Y, Chen XY, Xu Y. Comprehensive analysis of the gut microbiota in patients with chronic obstructive pulmonary disease of varying severity-A prospective, observational study. Heliyon. 2024 Jun 15;10(11):e31512. The authors declare that they have no known competing financial interests or personal relationships that could have appeared to influence the work reported in this paper. Epub 20240519. doi:10.1016/j.heliyon.2024.e31512. Cited in: Pubmed; PMID 38845997.

53. YANG Yan ZT, WANG Yang, ZHU Qin, MA Xiuqin. Correlation of intestinal microecological environment and lung function with blood gas analysis in patients with acute exacerbation of chronic obstructive pulmonary disease. J Cl in Pathol Res. 2022;42(3). doi:10.3978/j.issn.2095-6959.2022.03.008.

54. Deng S. Observation on the status of intestinal flora in patients with chronic obstructive pulmonary disease at stable phase and the efficacy of probiotics [master]: Jiangsu University; 2021. Cnki.

55. LUO Yong WY, HAN Feng-feng, XU Wei-guo. Changes of bifidobacterium and lactobacillus in gut of patients with COPD and their correlation with acute exacerbation frequency. Journal of Clinical Pulmonary Medicine. 2013;18(08):1366-1367. doi:10.3969 / j.issn.1009-6663.2013.08.004.

56. Junying Z. Characteristics of intestinal microflora in patients with chronic obstructive pulmonary disease and its correlation with inflammatory indexes and pulmonary function. Journal of Clinical Medicine in Practice. 2019;23(24):51-54. doi:10.7619 / jcmp.201924016.

57. Zhang M, Ding G, Dai B. Characteristics of intestinal flora in patients with AECOPD and the predictive efficacy of serum homocysteine and interleukin-6 on the intestinal flora disorder. Chinese Journal of Microecology. 2023;35(10):1187-1191. doi:10.13381/j.cnki.cjm.202310012.

86. Ma Y. Clinical study on the regulation of intestinal microflora microbial intervention on the stable phase of chronic obstructive pulmonary disease in the elderly [master]: Gansu University of Traditional Chinese Medicine; 2019. Cnki.

87. Zolnikova OY, Potskhverashvili ND, Kudryavtseva AV, Krasnov GS, Guvatova ZG, Truhmanov AS, Kokina NI, Ivashkin VT. [Changes in gut microbiota with bronchial asthma]. Ter Arkh. 2020 Apr 27;92(3):56-60. Epub 20200427. doi:10.26442/00403660.2020.03.000554. Cited in: Pubmed; PMID 32598794.

88. Burke DG, Fouhy F, Harrison MJ, Rea MC, Cotter PD, O'Sullivan O, Stanton C, Hill C, Shanahan F, Plant BJ, Ross RP. The altered gut microbiota in adults with cystic fibrosis. BMC Microbiol. 2017 Mar 9;17(1):58. Epub 20170309. doi:10.1186/s12866-017-0968-8. Cited in: Pubmed; PMID 28279152.

**Supplementary Table 3. Assessment of risk of bias in non-interventional comparative included studies.**

| **Estimated risk of bias in included comparative studies** | | | | | | | | |
| --- | --- | --- | --- | --- | --- | --- | --- | --- |
| **Study** | **Confounding** | **Selection** | **Measurement of Exposure** | **Missing data** | **Measurement of outcome** | **Reporting** | **Post-exposure intervention** | **Overall** |
| Chiyong Chen et al 2023 | **Moderate** | **Low** | **Low** | **Low** | **Low** | **Low** | **Low** | **Low** |
| Xiaoli Chen et al 2021 | **Moderate** | **Low** | **Low** | **Low** | **Moderate** | **Low** | **Low** | **Some concerns** |
| Yuexuan Chen et al 2018 | **Moderate** | **Low** | **Low** | **Low** | **Moderate** | **Low** | **Low** | **Some concerns** |
| Meng Zhang et al 2023 | **Moderate** | **Low** | **Low** | **Low** | **Low** | **Low** | **Low** | **Some concerns** |
| Zhouli Dai et al 2020 | **Serious** | **Low** | **Low** | **Low** | **Low** | **Low** | **Low** | **High** |
| Sumin Deng et al 2021 | **Low** | **Low** | **Low** | **Low** | **Low** | **Low** | **Low** | **Low** |
| Yonghong Wu et al 2021 | **Serious** | **Low** | **Low** | **Low** | **Low** | **Low** | **Low** | **High** |
| Yuting Kang et al 2022 | **Moderate** | **Low** | **Low** | **Low** | **Low** | **Low** | **Low** | **Low** |
| Qianqian Jiao et al 2022 | **Moderate** | **Low** | **Low** | **Low** | **Moderate** | **Low** | **Low** | **Some concerns** |
| Yong Luo et al.2013 | **Moderate** | **Low** | **Low** | **Low** | **Low** | **Low** | **Low** | **Some concerns** |
| Yukun Ma et al 2019 | **Low** | **Low** | **Low** | **Low** | **Moderate** | **Low** | **Low** | **Some concerns** |
| Junying Zhang et al 2019 | **Moderate** | **Low** | **Low** | **Low** | **Low** | **Low** | **Low** | **Some concerns** |
| Yu Zhang et al 2021 | **Low** | **Low** | **Low** | **Low** | **Moderate** | **Low** | **Low** | **Some concerns** |
| Yimeng Xu et al 2022 | **Moderate** | **Low** | **Low** | **Low** | **Low** | **Low** | **Low** | **Some concerns** |
| Jing Zou et al 2023 | **Low** | **Low** | **Low** | **Low** | **Low** | **Low** | **Low** | **Low** |
| Yang Liu et al 2022 | **Low** | **Low** | **Low** | **Low** | **Low** | **Low** | **Low** | **Low** |
| Jiali Yan et al 2024 | **Low** | **Low** | **Low** | **Low** | **Low** | **Low** | **Low** | **Low** |
| Naijian Li et al 2021 | **Serious** | **Low** | **Low** | **Low** | **Low** | **Low** | **Low** | **Low** |
| Wenxing Yong et al 2020 | **Moderate** | **Low** | **Low** | **Low** | **Moderate** | **Low** | **Low** | **Some concerns** |
| Yan Yang et al 2022 | **Low** | **Low** | **Low** | **Low** | **Low** | **Low** | **Low** | **Low** |
| Kate L. Bowerman et al 2020 | **Low** | **Low** | **Low** | **Low** | **Low** | **Low** | **Low** | **Low** |
| Yaoxin Chen et al 2023 | **Low** | **Low** | **Low** | **Low** | **Moderate** | **Low** | **Low** | **Some concerns** |
| Ming Liu et al 2024 | **Low** | **Moderate** | **Low** | **Low** | **Low** | **Low** | **Low** | **Some concerns** |
| Hafiz Muhammad Ishaq et al 2018 | **Moderate** | **Moderate** | **Low** | **Low** | **Low** | **Low** | **Low** | **Some concerns** |
| Yang Liu et al 2022 | **Low** | **Low** | **Low** | **Low** | **Low** | **Low** | **Low** | **Low** |
| О.Yu. Zolnikova et al 2020 | **Low** | **Moderate** | **Low** | **Low** | **Low** | **Low** | **Low** | **Some concerns** |
| Xiaoling Zou et al 2021 | **Low** | **Low** | **Low** | **Low** | **Low** | **Low** | **Low** | **Low** |
| D. G. Burke, F et al 2017 | **Moderate** | **Moderate** | **Low** | **Low** | **Low** | **Low** | **Low** | **Some concerns** |

**Supplementary Table 4 β diversity in CLD patients.**

**4.1 β diversity in COPD patients.**

| **Disease** | **Study** | **Method** | **Result** |
| --- | --- | --- | --- |
| COPD | Chiyong Chen et al 2023 | Bray-Curtis dissimilarity index | Distinct |
|  | Xiaoli Chen et al 2021 | Weighted UniFrac | Distinct |
|  | Yuexuan Chen et al 2018 | Non metric multidi-mensional scaling (NMDS) | Distinct |
|  | Meng Zhang et al 2023 | N/A | N/A |
|  | Zhouli Dai et al 2020 | principal component analysis (PCA) | Distinct |
|  | Sumin Deng et al 2021 | N/A | N/A |
|  | Yonghong Wu et al 2021 | PCA | Distinct |
|  | Yuting Kang et al 2022 | PCA | Distinct |
|  | Qianqian Jiao et al 2022 | N/A | N/A |
|  | Yong Luo et al.2013 | N/A | N/A |
|  | Yukun Ma et al 2019 | N/A | N/A |
|  | Junying Zhang et al 2019 | N/A | N/A |
|  | Yu Zhang et al 2021 | Principal Coordinate Analysis (PCoA) | Distinct |
|  | Yimeng Xu et al 2022 | PCA | Indistinct |
|  | Jing Zou et al 2023 | PCoA | Distinct |
|  | Yang Liu et al 2022 | N/A | N/A |
|  | Jiali Yan et al 2024 | Partial Least Squares Discriminant Analysis (PLS-DA) | Distinct |
|  | Naijian Li et al 2021 | PCoA | Distinct |
|  | Wenxing Yong et al 2020 | NMDS | Distinct |
|  | Yan Yang et al 2022 | N/A | N/A |
|  | Kate L. Bowerman et al 2020 | PCA | Distinct |

**4.2 β diversity in asthma or CF patients.**

| **Disease** | **Study** | **Method** | **Result** |
| --- | --- | --- | --- |
| **Asthma** | Yaoxin Chen et al 2023 | PLS-DA | Distinct |
|  | Ming Liu et al 2024 | NMDS | Distinct |
|  | Hafiz Muhammad Ishaq et al 2018 | N/A | N/A |
|  | Yang Liu et al 2022 | N/A | N/A |
|  | О.Yu. Zolnikova et al 2020 | N/A | N/A |
|  | Xiaoling Zou et al 2021 | PCA, PLS-DA | Distinct |
|  |  |  |  |
| **CF** | D. G. Burke, F et al 2017 | PCA | Distinct |

**Supplementary Table 5. Differentially abundant microbial taxa.**

**5.1 COPD**

| **Study** | **Phylum level** | | **Family level** | | **Genus level** | |
| --- | --- | --- | --- | --- | --- | --- |
|  | **Higher** | **Lower** | **Higher** | **Lower** | **Higher** | **Lower** |
| Chiyong Chen et al 2023 | N/A | N/A | N/A | N/A | Not Found | Bacteroides, Prevotella |
| Xiaoli Chen et al 2021 | Firmicutes, Actinobacteria | Bacteroidetes | N/A | N/A | Enterococcus, Lactobacillus | Bacteroides |
| Yuexuan Chen et al 2018 | N/A | N/A | N/A | N/A | N/A | N/A |
| Meng Zhang et al 2023 | N/A | N/A | N/A | N/A | Not Found | Bifidobacterium and Lactobacillus |
| Zhouli Dai et al 2020 | Firmicutes | Bacteroidetes | N/A | N/A | N/A | N/A |
| Sumin Deng et al 2021 | N/A | N/A | N/A | N/A | Enterococcus faecalis, Enterococcus faecium | Bifidobacterium and Lactobacillus |
| Yonghong Wu et al 2021 | Bacteroidetes, Proteobacteria | Firmicutes, Actinobacteria | N/A | N/A | Bacteroides, Lachnoclostridium, and Parabacteroides | [Eubacterium]_hallii_group, Anaerostipes, Bifidobacterium, Blautia, Fusicatenibacter, and Lachnospira |
| Yuting Kang et al 2022 | N/A | N/A | N/A | N/A | N/A | N/A |
| Qianqian Jiao et al 2022 | N/A | N/A | N/A | N/A | Bacteroides | Faecalibactrium |
| Yong Luo et al.2013 | N/A | N/A | N/A | N/A | Not Found | Bifidobacterium and Lactobacillus |
| Yukun Ma et al 2019 | N/A | N/A | N/A | N/A | Eubacterium rectale | Bacteroides thetaiotaomicron |
| Junying Zhang et al 2019 | N/A | N/A | N/A | N/A | Not Found | Bifidobacteria, lactobacillus |
| Yu Zhang et al 2021 | Proteobacteria | Firmicutes | N/A | N/A | Coprobacter, Bacteroides | Prevotella, Rumen bacteria, Eubacillus |
| Yimeng Xu et al 2022 | Not Found | Not Found | N/A | N/A | Not Found | Prevotella,Massilia, Megasphaera |
| Jing Zou et al 2023 | Not Found | Firmicutes, Proteobacteria, Actinobacteria | Enterococcaceae | Ruminococcacea, Clostridiaceae, | Enterococcus, Lachnoclostridium | Faeacalibacterium, Blautia, Anaerostipes |
| Yang Liu et al 2022 COPD | Faecalicatena, Oscillibacter, Lawsonibacter, Flavonifractor, Streptomyces, Absiella, Pseudomonas_E, Anaerotruncus, Hungatella, Pseudoflavonifractor | Lachnospira, Eubacterium_F, Coprococcus, Ruminococcus_C, Coprococcus_A | N/A | N/A | N/A | N/A |
| Jiali Yan et al 2024 | Subdoligranulum, Lachnoclostridium, Escherichia-Shigella, Enterococcu | Bacteroides,Roseburia,Roseburia | N/A | N/A | N/A | N/A |
| Naijian Li et al 2021 | Enterococcus, Prevotella | Bacteroidetes | Prevotellaceae | Bacteroidaceae, Fusobacteriaceae | N/A | N/A |
| Wenxing Yong et al 2020 | Bacteroidetes | Firmicutes | N/A | N/A | Lachnospira, Rothia, Coprococcus | Bifidobacterium |
| Yan Yang et al 2020 | N/A | N/A | N/A | N/A | Enterococcus faecalis and Enterococcus faecium | Bifidobacterium and Lactobacillus |
| Kate L. Bowerman et al 2020 | N/A | N/A | N/A | N/A | Streptococcus, Rothia, Romboutsia and Intestinibacter | Bacteroides, Roseburia and Lachnospira |

**5.2 Asthma and CF**

| **Study** | **Phylum level** | | **Family level** | | **Genus level** | |
| --- | --- | --- | --- | --- | --- | --- |
|  | **Higher** | **Lower** | **Higher** | **Lower** | **Higher** | **Lower** |
| Yaoxin Chen et al 2023 | Actinobacteria | Cyanobacteria | Sutterellaceae | Not Found | Blautia | Not Found |
| Ming Liu et al 2024 | Bacteroidetes | Chloroflexi | N/A | N/A | Streptomyces，[Eubacterium]_ventriosum_group，Butyricicoccus，Agathobacter | Faecalibacterium，Roseburia，Alistipes，Sphingomonas，Dorea |
| Hafiz Muhammad Ishaq et al 2018 | N/A | N/A | N/A | N/A | N/A | N/A |
| Yang Liu et al 2022 | Bacteroides, Faecalibacterium, Agathobacter, Blautia_A, Roseburia, Fusicatenibacter, Faecalicatena, Dorea, Oscillibacter, Lawsonibacter | Phil1 | N/A | N/A | N/A | N/A |
| О.Yu. Zolnikova et al 2020 | N/A | N/A | N/A | N/A | Faecalibacterium, Rikenellaceae, Alistipes | Grammaproteobacteria, Enterobacteriaceae, Escherichia/Shigella |
| Xiaoling Zou et al 2021 | Not Found | Not Found | N/A | N/A | N/A | N/A |
| D. G. Burke, F et al 2017 ^a^ | Actinobacteria and Firmicutes | Bacteroidetes, Proteobacteria, Cyanobacteria, Verrucomicrobia, RF3, Tenericutes, and Lentisphaerae | Not Found | Alcaligenaceae, Prevotellaceae, Bifidobacteriaceae and Peptococcaceae | Enterococcus, Bacteroides, Leuconostoc | Roseburia, Prevotella, Odoribacter, Faecalibacterium and Bifidobacterium |

**Note: a, cystic fibrosis (CF); N/A: not report.**

**Supplementary Table 6. Publication bias assessment for the alpha diversity mete-analyses by egger test.**

| Disease | Index | Standard Error | t | P value | 95% CI |
| --- | --- | --- | --- | --- | --- |
| COPD | Chao1 | 7.266529 | -0.42 | 0.695 | -21.69961~15.6588 |
|  | Shannon | 1.674807 | -1.51 | 0.192 | -6.831415~1.779044 |
|  | Simpson | 2.394079 | 1.08 | 0.393 | -7.713052~12.88873 |
|  | Firmicutes | 6.238105 | -1.24 | 0.342 | -34.55176~19.12904 |
|  | Bacteroidetes | 4.290828 | 1.15 | 0.37 | -13.53314~23.39075 |
|  | Proteobacteria | 4.639543 | 0.32 | 0.78 | -18.47963~21.44506 |
|  | Actinobacteria | 7.107674 | -1.14 | 0.374 | -38.65178~22.51192 |
|  | lactobacillus | 2.904681 | 1.36 | 0.266 | -5.284701~13.20328 |
|  | bifidobacterium | 3.098077 | 1.89 | 0.156 | -7.501143~29.32656 |
| Asthma | Shannon | 2.259453 | -0.65 | 0.583 | -11.18812~8.255166 |

**Supplementary Table 7. The crude values of the different indicators in the COPD or asthma patients.**

**7.1 The crude values of ACE index in the COPD patients.**

| Author | Year | m1 | s1 | n1 | m2 | s2 | n2 |
| --- | --- | --- | --- | --- | --- | --- | --- |
| Qianqian Jiao | 2022 | 559 | 320 | 22 | 749 | 369 | 21 |
| Yu Zhang | 2021 | 333.27 | 140.3 | 11 | 382.89 | 125.7 | 9 |

**7.2 The crude values of chao1 index in the COPD patients.**

| Author | Year | m1 | s1 | n1 | m2 | s2 | n2 |
| --- | --- | --- | --- | --- | --- | --- | --- |
| Yuexuan Chen | 2018 | 834.418 | 194.447 | 58 | 348.844 | 98.2119 | 30 |
| Zhouli Dai | 2020 | 877.93 | 160.58 | 101 | 802.5 | 158.98 | 73 |
| Yonghong Wu | 2020 | 331.53 | 138.86 | 58 | 445.43 | 105.25 | 22 |
| Wenxing Yong | 2020 | 674.16 | 439.36 | 58 | 807.49 | 402.9 | 30 |
| Yu Zhang | 2021 | 328.27 | 138.98 | 11 | 382.33 | 126.7 | 9 |
| Xiaoli Chen | 2021 | 418.06 | 121.16 | 59 | 692.129 | 86.0359 | 30 |
| Qianqian Jiao | 2022 | 534 | 300 | 22 | 721 | 361 | 21 |

**7.3 The crude values of Shannon index in the COPD patients.**

| Author | Year | m1 | s1 | n1 | m2 | s2 | n2 |
| --- | --- | --- | --- | --- | --- | --- | --- |
| Yonghong Wu | 2020 | 4.68 | 1.1 | 58 | 5.56 | 0.63 | 22 |
| Qianqian Jiao | 2020 | 5.45 | 1.29 | 22 | 5.94 | 1.84 | 21 |
| Xiaoli Chen | 2021 | 4.16 | 3.2 | 59 | 5.6 | 0.7 | 30 |
| Yu Zhang | 2021 | 2.71 | 0.67 | 11 | 3.07 | 0.84 | 9 |
| Yuting Kang | 2022 | 1.73 | 0.5285 | 10 | 2.15 | 0.2464 | 10 |
| Jing Zou | 2023 | 2.24 | 0.8413 | 15 | 2.27 | 0.6012 | 5 |
| Chiyong Chen | 2023 | 2.34 | 0.2766 | 80 | 2.25 | 0.3544 | 80 |

**7.4 The crude values of Simpson index in the COPD patients**

| Author | Year | m1 | s1 | n1 | m2 | s2 | n2 |
| --- | --- | --- | --- | --- | --- | --- | --- |
| Yonghong Wu | 2020 | 0.9 | 0.09 | 58 | 0.95 | 0.03 | 22 |
| Yu Zhang | 2021 | 0.2 | 1.11 | 11 | 0.17 | 0.16 | 9 |
| Qianqian Jiao | 2022 | 0.91 | 0.07 | 22 | 0.88 | 0.2 | 21 |
| Jing Zou | 2023 | 0.8455 | 0.1043 | 15 | 0.8334 | 0.1001 | 5 |

**7.5 The crude values of chao1 index in the asthma patients.**

| Author | Year | m1 | s1 | n1 | m2 | s2 | n2 |
| --- | --- | --- | --- | --- | --- | --- | --- |
| Yaoxin Chen | 2023 | 367.79 | 35.817 | 26 | 357.86 | 51.82 | 11 |
| Ming Liu | 2024 | 1071.178 | 22.3496 | 14 | 1244.4496 | 190.2079 | 15 |

**7.6 The crude values of Shannon index in the asthma patients.**

| Author | Year | m1 | s1 | n1 | m2 | s2 | n2 |
| --- | --- | --- | --- | --- | --- | --- | --- |
| Hafiz Muhammad Ishaq | 2018 | 2 | 0.31 | 15 | 2.27 | 0.21 | 5 |
| Xiaoling Zou | 2021 | 4.7509 | 1.179 | 47 | 5.1278 | 0.9641 | 20 |
| Yaoxin Chen | 2023 | 3.01 | 0.4346 | 26 | 2.95 | 0.72 | 11 |
| Ming Liu | 2024 | 5.448 | 0.5691 | 14 | 5.7021 | 1.3681 | 15 |

**7.7 The crude values of Simpson index in the asthma patients**

| Author | Year | m1 | s1 | n1 | m2 | s2 | n2 |
| --- | --- | --- | --- | --- | --- | --- | --- |
| Yaoxin Chen | 2023 | 0.14 | 0.0756 | 26 | 0.15 | 0.11 | 11 |
| Ming Liu | 2024 | 0.9125 | 0.0177 | 14 | 0.9257 | 0.0292 | 15 |

**7.8 The crude values of Bacteroides in the COPD patients**.

| Author | Year | m1 | s1 | n1 | m2 | s2 | n2 |
| --- | --- | --- | --- | --- | --- | --- | --- |
| Yonghong Wu | 2020 | 36.73 | 4.94 | 58 | 29.06 | 3.88 | 22 |
| Wenxing Yong | 2020 | 40.71 | 4.88 | 58 | 36.3 | 3.9 | 30 |
| Zhouli Dai | 2020 | 49.04 | 15.94 | 101 | 50.5 | 14.3 | 73 |
| Jing Zou | 2023 | 45.6658 | 34.4222 | 15 | 25.5381 | 20.2573 | 5 |

**7.9 The crude values of Firmicutes in the COPD patients.**

| Author | Year | m1 | s1 | n1 | m2 | s2 | n2 |
| --- | --- | --- | --- | --- | --- | --- | --- |
| Yonghong Wu | 2020 | 51.22 | 5.65 | 58 | 51.48 | 4.95 | 22 |
| Wenxing Yong | 2020 | 28.91 | 4.64 | 58 | 50.7 | 7.9 | 30 |
| Zhouli Dai | 2020 | 41.56 | 16.04 | 101 | 40 | 15.2 | 73 |
| Jing Zou | 2023 | 36.0302 | 26.4786 | 15 | 60.1525 | 7.9582 | 5 |

**7.10 The crude values of Proteobacteria in the COPD patients.**

| Author | Year | m1 | s1 | n1 | m2 | s2 | n2 |
| --- | --- | --- | --- | --- | --- | --- | --- |
| Yonghong Wu | 2020 | 0.044 | 0.0731 | 58 | 0.0146 | 0.0103 | 22 |
| Wenxing Yong | 2020 | 15.3879 | 4.3146 | 58 | 10.18181818 | 1.75 | 30 |
| Zhouli Dai | 2020 | 4.3793 | 4.2548 | 101 | 4.2868 | 2.6016 | 73 |
| Jing Zou | 2023 | 1.3723 | 2.6479 | 15 | 2.2982 | 4.7026 | 5 |

**7.11 The crude values of Actinomyces in the COPD patients.**

| Author | Year | m1 | s1 | n1 | m2 | s2 | n2 |
| --- | --- | --- | --- | --- | --- | --- | --- |
| Yonghong Wu | 2020 | 6.04 | 1.04 | 58 | 10.5 | 1.68 | 22 |
| Wenxing Yong | 2020 | 3.06 | 1.33 | 58 | 6.4 | 1 | 30 |
| Zhouli Dai | 2020 | 0.36 | 0.42 | 101 | 0.33 | 0.44 | 73 |
| Jing Zou | 2023 | 8.2658 | 15.0045 | 15 | 6.1349 | 4.7026 | 5 |

**7.12 The crude values of Bifidobacterium in the COPD patients.**

| Author | Year | m1 | s1 | n1 | m2 | s2 | n2 |
| --- | --- | --- | --- | --- | --- | --- | --- |
| Yong Luo | 2013 | 13.03 | 1.185 | 40 | 13.418 | 1.411 | 40 |
| Junying Zhang | 2019 | 13.04 | 1.07 | 104 | 14.79 | 1.41 | 80 |
| Sumin Deng | 2021 | 6.84 | 1.39 | 80 | 7.23 | 1.67 | 20 |
| Yan Yang | 2022 | 6.2 | 1.04 | 96 | 7.31 | 1.56 | 86 |
| Meng Zhang | 2023 | 7.14 | 1.22 | 96 | 9.85 | 2.28 | 88 |

**7.13 The crude values of Lactobacillus in the COPD patients.**

| Author | Year | m1 | s1 | n1 | m2 | s2 | n2 |
| --- | --- | --- | --- | --- | --- | --- | --- |
| Yong Luo | 2013 | 10.793 | 1.166 | 40 | 11.43 | 2.23 | 40 |
| Junying Zhang | 2019 | 10.74 | 1.17 | 104 | 11.87 | 2.01 | 80 |
| Sumin Deng | 2021 | 6.165 | 1.8671 | 80 | 7.49 | 1.66 | 20 |
| Yan Yang | 2022 | 5.59 | 1.11 | 96 | 7.51 | 2.02 | 86 |
| Meng Zhang | 2023 | 7.96 | 1.52 | 96 | 9.05 | 2.11 | 88 |

Note: n1, m1, s1: number of cases, mean and standard deviation in the experimental group; n2, m2, s2: number of cases, mean and standard deviation in the control group.

**Supplementary Figure 1. Sensitivity analysis.**

**Figure 1.1 Sensitivity analysis of α-diversity in COPD.**


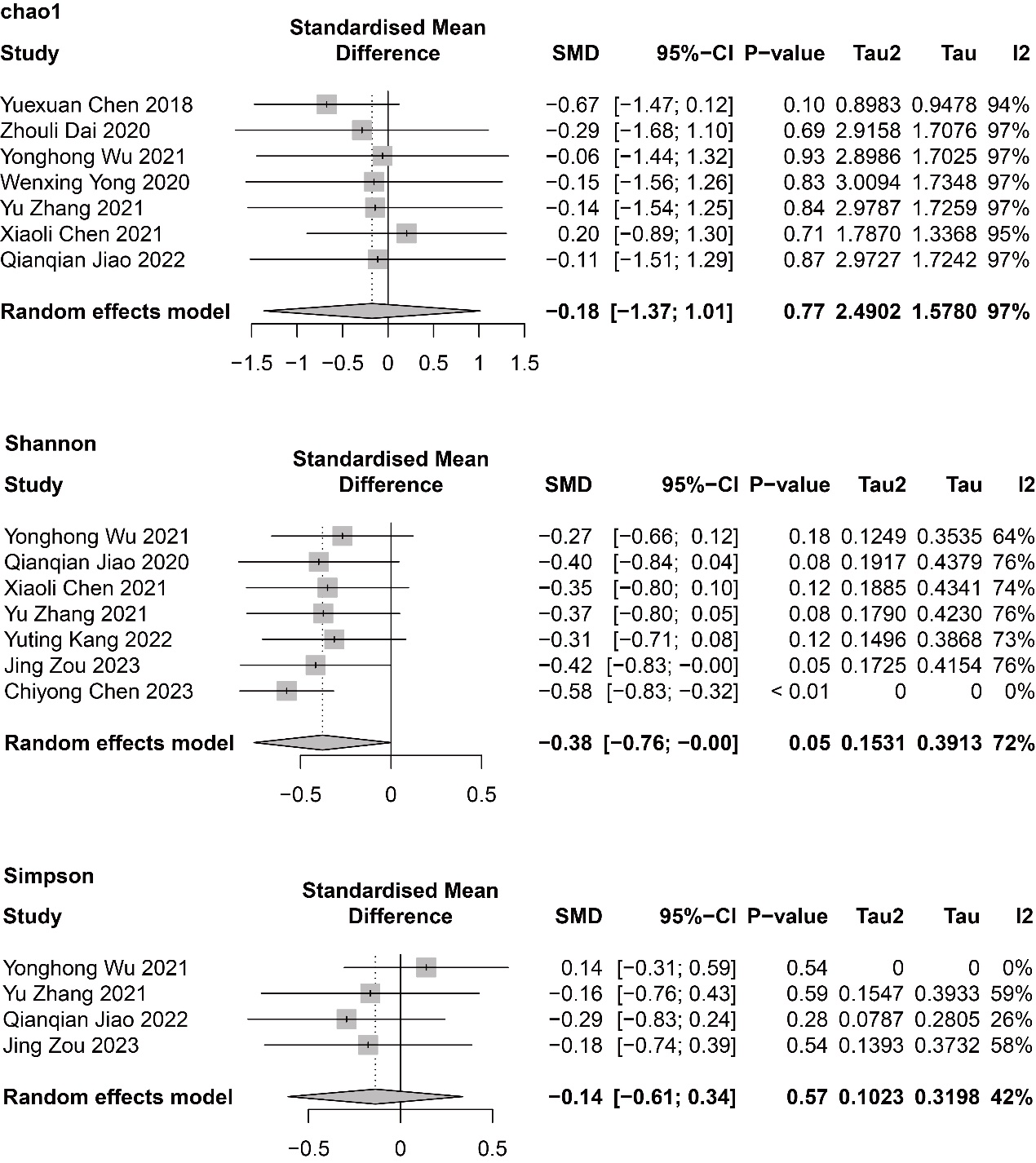


**Figure 1.2 Sensitivity analysis of α-diversity in asthma.**


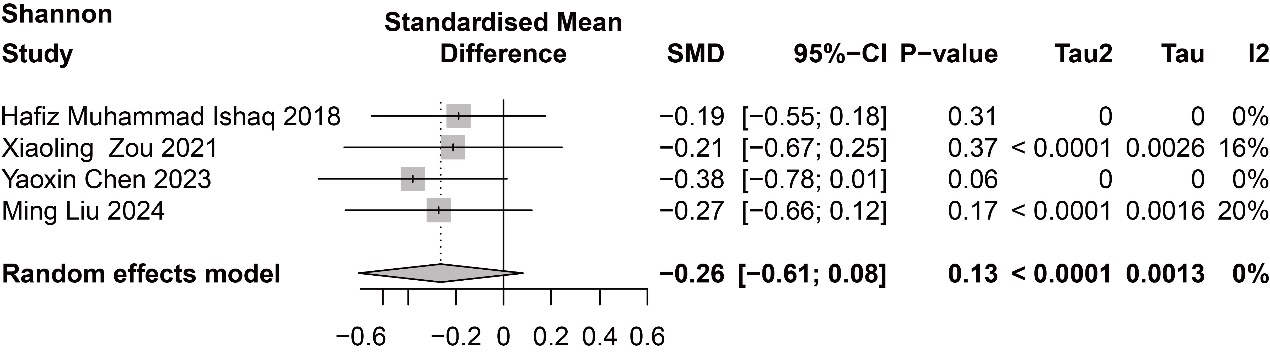


**Figure 1.3 Sensitivity analysis of phylum level in COPD.**


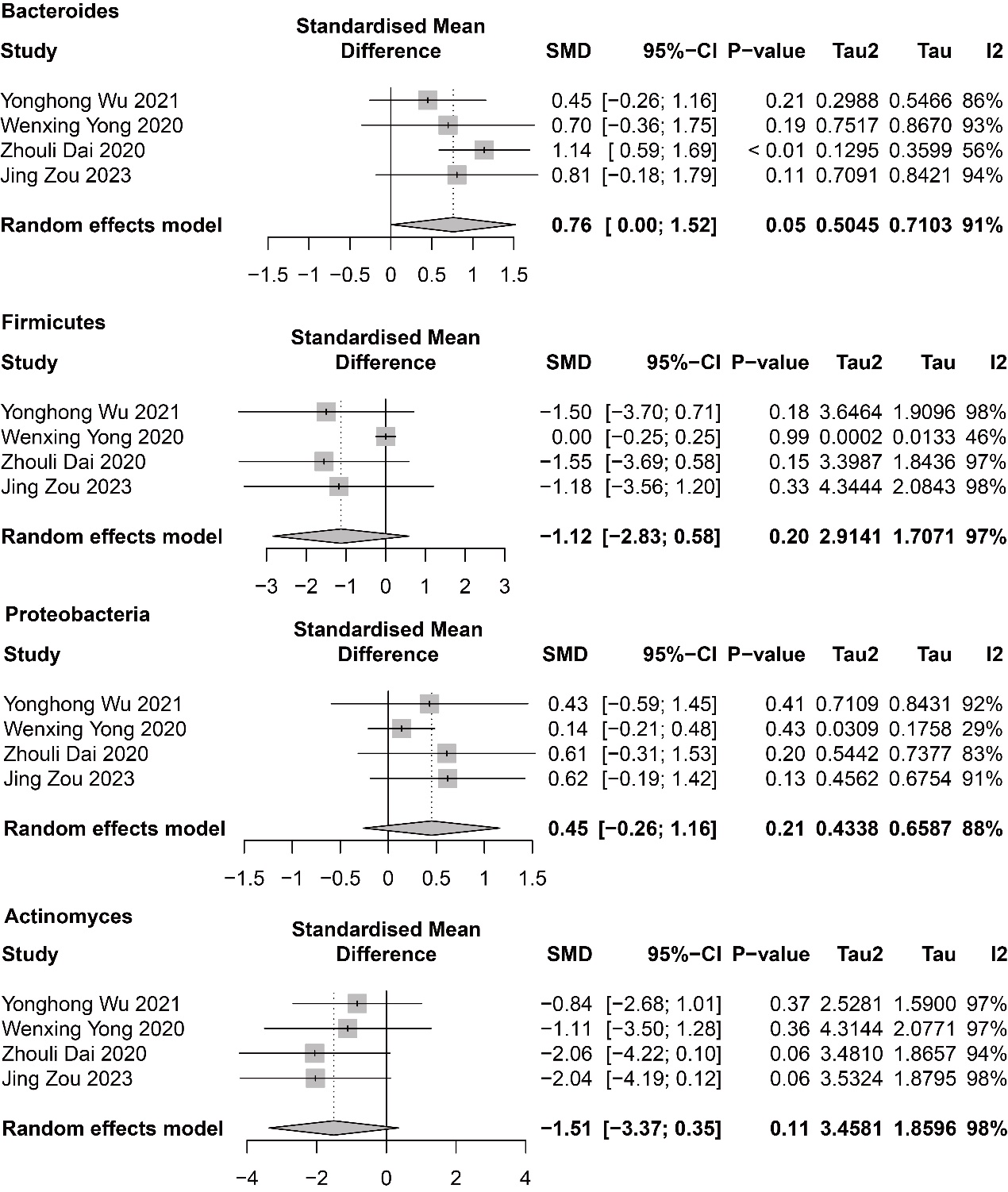


**Figure 1.4 Sensitivity analysis of Bifidobacterium and Lactobacillus in COPD.**
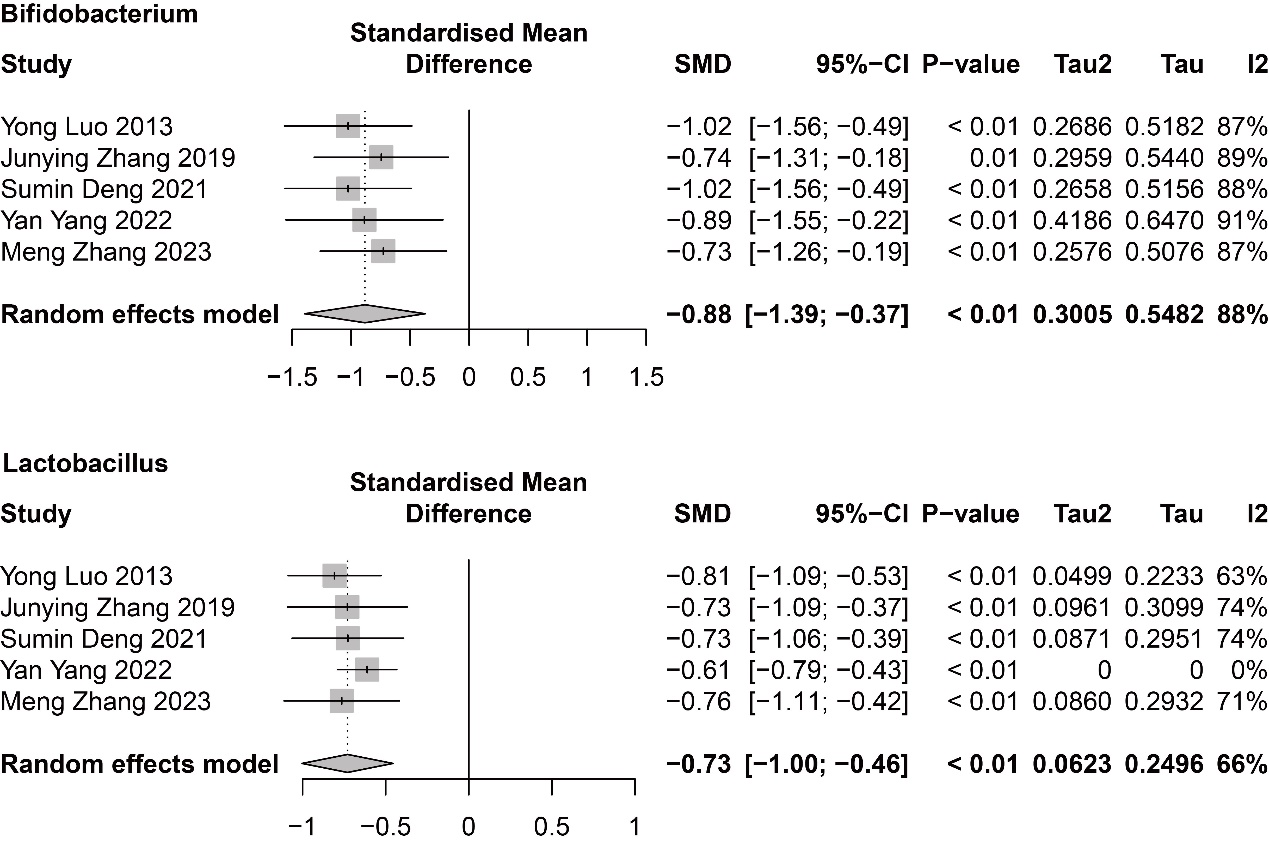


**Supplementary Figure 2.** Subgroup analysis of chao1 in the COPD patients according to the traditional Chinese medicine treatment.


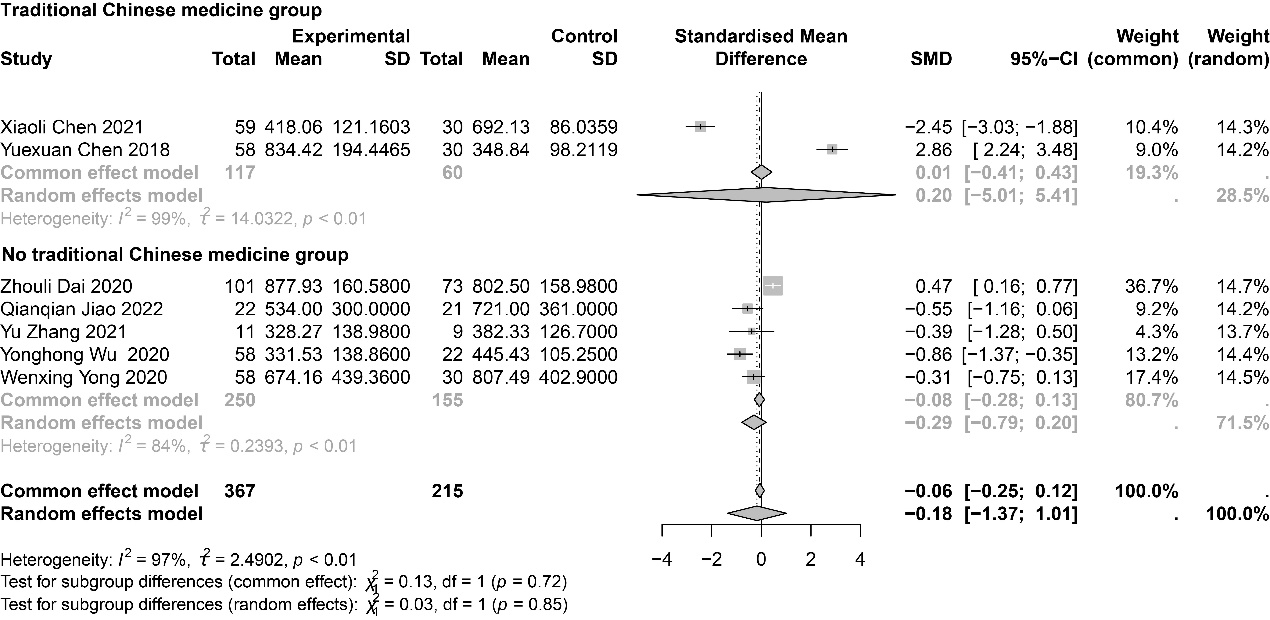

Supplement: Supplementary file 1 [file DataSheet1.docx]
